# VEGFR2 signaling drives meningeal vascular regeneration upon head injury

**DOI:** 10.1038/s41467-020-17545-2

**Published:** 2020-07-31

**Authors:** Bong Ihn Koh, Hyuek Jong Lee, Pil Ae Kwak, Myung Jin Yang, Ju-Hee Kim, Hyung-Seok Kim, Gou Young Koh, Injune Kim

**Affiliations:** 1 KI for Bio-century, Korea Advanced Institute of Science and Technology (KAIST), Daejeon, 34141 Republic of Korea; 2 Center for Vascular Research, Institute for Basic Science (IBS), Daejeon, 34141 Republic of Korea; 3 Graduate School of Medical Science and Engineering, Korea Advanced Institute of Science and Technology, Daejeon, 34141 Republic of Korea; 4 Department of Forensic Medicine, Chonnam National University Medical School, Gwangju, 61463 Republic of Korea

**Keywords:** Mechanisms of disease, Angiogenesis, Regeneration and repair in the nervous system

## Abstract

Upon severe head injury (HI), blood vessels of the meninges and brain parenchyma are inevitably damaged. While limited vascular regeneration of the injured brain has been studied extensively, our understanding of meningeal vascular regeneration following head injury is quite limited. Here, we identify key pathways governing meningeal vascular regeneration following HI. Rapid and complete vascular regeneration in the meninges is predominantly driven by VEGFR2 signaling. Substantial increase of VEGFR2 is observed in both human patients and mouse models of HI, and endothelial cell-specific deletion of *Vegfr2* in the latter inhibits meningeal vascular regeneration. We further identify the facilitating, stabilizing and arresting roles of Tie2, PDGFRβ and Dll4 signaling, respectively, in meningeal vascular regeneration. Prolonged inhibition of this angiogenic process following HI compromises immunological and stromal integrity of the injured meninges. These findings establish a molecular framework for meningeal vascular regeneration after HI, and may guide development of wound healing therapeutics.

## Abstract (web summary)

Severe head injury results in critical damage of blood vessels of the meninges and brain parenchyma. Here, the authors describe key pathways governing meningeal vascular regeneration following head injury, characterizing the differential roles of VEGFR2, Tie2, Dll4 and PDGFRβ signaling.

## Introduction

Traumatic brain injuries (TBIs) arising from various accidents, ranging from vehicle-related collisions to sports injuries, are the most frequent form of brain injury in humans^1,2^. TBI may cause a wide range of health issues from behavioral changes to pronounced cognitive dysfunction^3–5^. As TBI causes injury to both the brain parenchyma (hereafter, referred to as “brain”) and neighboring meningeal blood vessels (BVs), such injuries are better considered as head injuries (HIs) with respect to all the cranial tissues affected by the physical impact.

The meninges are composed of the dura mater, arachnoid membrane, subarachnoid space, and pia mater, all of which protect the brain^6,7^. The dura mater is highly vascularized with precapillary arterioles, capillary beds, and postcapillary venules^8,9^, of which the latter two vessel types support immune cell trafficking and surveillance for the protection of the central nervous system^10^.

Although a plethora of studies has investigated the molecular mechanisms underlying vascular responses in the brain following TBI^11–13^, responses to such inevitable injuries in the neighboring meninges remain largely unexplored. A recent report indicates that, upon compression injury to the skull, injured meningeal BVs undergo rapid regeneration with the aid of different macrophage subsets^14^. Although this previous study demonstrates the myeloid cells’ ability to break down physical obstructions in the angiogenic path, it does not identify that fundamental angiogenic pathways contribute to the rapid regeneration of injured meningeal BVs.

In this study, we investigate which underlying molecular and cellular mechanisms drive vascular regeneration in the dura mater. We provide a comprehensive molecular characterization of dura mater BVs both in homeostasis and following vascular injury, highlighting fundamental differences between dura mater and brain endothelial cells (ECs) and thereby suggesting differential organotypic treatment for HI care.

## Results

### Dynamic vascular regeneration occurs in dura mater after PTI

**Fig. 1:**
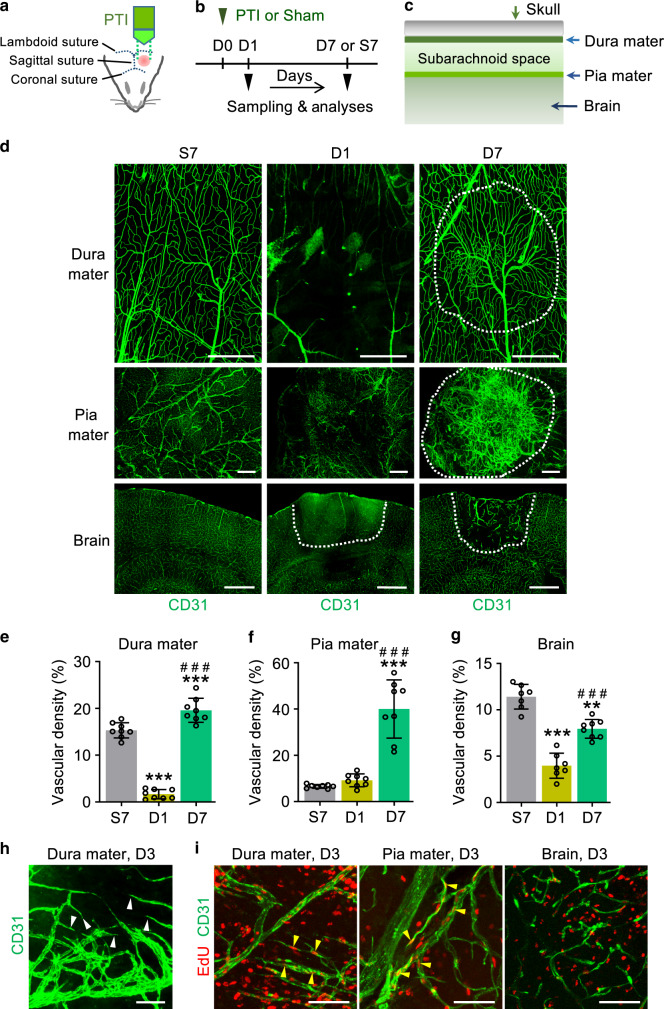
Differential vascular regeneration upon PTI in meninges and brain. **a** Schematic diagram depicting PTI onto mouse head as HI model. Pink circle indicates Rose Bengal activated by white light (3 mW). **b** Diagram depicting the experimental scheme for PTI or sham (Sham, S) operation, sampling, and analyses at indicated time points; D0, D1, and D7, day 0, 1, and 7 after PTI; S7, day 7 after sham operation. **c** Schematic diagram depicting layers of meninges between skull and brain. **d** Representative images of CD31 + BVs in dura mater, pia mater, and brain at S7, D1, and D7 in adult mice. Each white dotted-lined circle or line indicates putative injury area. Note rapid and organized vascular regeneration in the dura mater, whereas there is excessive or insufficient and disorganized vascular regeneration in the pia mater or brain. Scale bars, 500 µm. **e**–**g** Comparisons of CD31+ vascular densities in the indicated tissues. Each dot indicates a value from one mouse and *n* = 7 (Brain S7, Brain D1), *n* = 8 (all other groups) mice/group from three independent experiments. Vertical bars indicate mean ± SD. ***P < 0.001 versus S7 and ^###^*P* < 0.001 versus D1 by Kruskal–Wallis test. **h**, **i** Representative images of sprouting (white arrowheads) angiogenesis and EdU-incorporated EC proliferation (yellow arrowheads) at D3 in the indicated tissues of adult mice. Similar findings were observed in two independent experiments using *n* = 3–4 mice. Scale bars, 100 µm.

We employed photothrombotic injury (PTI)^15^ as an HI model to inflict vascular damage simultaneously onto the dura mater, pia mater, and brain of mice and compared the vascular changes in these compartments (Fig. 1a–c). Vascular responses to PTI were different for each compartment (Fig. 1d–g). In the dura mater, BVs re-vascularized avascular areas swiftly and almost completely regenerated to its original state at 7 days after PTI (D7) (Fig. 1d, e). Given that EdU-incorporated proliferative ECs formed typical sprouts at the vascular front of the recovering dura mater at D3 (Fig. 1h, i), vascular regeneration seemed to be mediated mainly by active sprouting angiogenesis. In the pia mater, the injured BVs undertook extensive vascular remodeling, including active sprouting angiogenesis highlighted by numerous vascular sprouts and proliferative ECs at D3 (Fig. 1i), which led to excessive vascular content with abnormally dilated BVs at D7 (Fig. 1d, f). In contrast, the brain vessels exhibited the poorest rate of regeneration, predominantly showing vascular collapse, substantial vascular dilation, and large avascular areas at D7 (Fig. 1d, g). Vascular sprouts and EdU-incorporated proliferative ECs were rarely found in the brain at D3 (Fig. 1i). Lectin perfusion analysis revealed that the recovered BVs exhibited functional blood flow by 80–90% and 92–98% at D7 and D14 in the dura mater (Supplementary Fig. 1a, b). In contrast, regeneration of blood perfusion was rarely seen in the brain (Supplementary Fig. 1c), highlighting the superior functional regeneration of dura mater BVs following PTI.

### PTI causes minor and transient vascular leakage in dura mater

Edema caused by vascular leakage and tissue death can cause severe complications in patients with TBI^16^. Evans Blue leakage analysis revealed minimal and transient vascular leakage at D3 and almost no vascular leakage thereafter in the dura mater (Supplementary Fig. 2a, d). In contrast, injured BVs of the pia mater and brain showed profound vascular leakage, particularly within the injury core, at D3 and D7 but sharply decreased over time. Nevertheless, this vascular leakage persisted at D28 (Supplementary Fig. 2b–d). Not only do these findings underscore the detrimental consequences of TBI in terms of brain edema, but they also highlight the superior regeneration potential of dura mater vascular integrity, in comparison to those in the pia mater and brain, after PTI.

### VEGFR2, Tie2, and Dll4 contribute to vascular regeneration

**Fig. 2:**
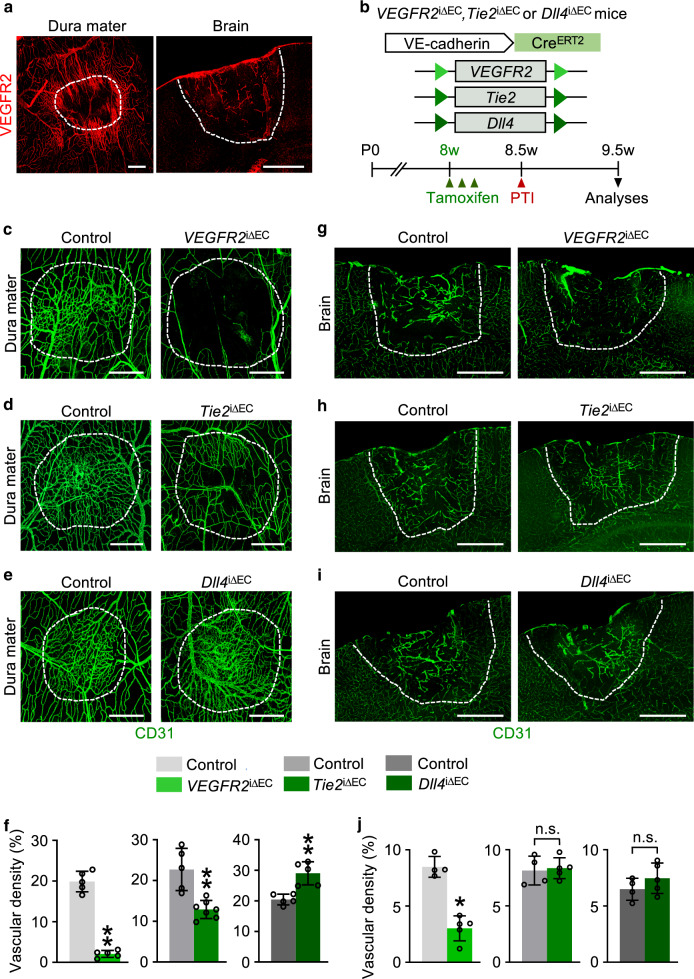
VEGFR2 signaling is critical for vascular regeneration in dura mater and brain after PTI. **a** Representative images of VEGFR2 upregulation in the surrounding BVs of putative injury areas (white dotted-lined circle or line) at D3 in adult mice. Similar findings were observed in four mice from two independent experiments. Scale bars, 500 µm. **b** Diagram depicting generation of *VEGFR2*^iΔEC^, *Tie2*^iΔEC^, or *Dll4*^iΔEC^ mice and EC-specific deletion of *VEGFR2*, *Tie2*, or *Dll4* in 8-week-old mice by i.p. injections of tamoxifen, PTI, and their analyses at 1 week after PTI. **c**–**j** Representative images and comparisons of CD31+BVs of putative injury areas (white dotted-lined circle or line) in the dura mater and brain at D7 in control and *VEGFR2*^iΔEC^, *Tie2*^iΔEC^, or *Dll4*^iΔEC^ mice. Scale bars, 500 µm. Each dot indicates a value from one mouse and *n* = 7 (Dura mater *Tie2*^iΔEC^), *n* = 4 (Brain controls), *n* = 5 (all other groups) mice/group from two independent experiments. Vertical bars indicate mean ± SD. *P < 0.05, **P < 0.01 versus control by two-tailed Mann–Whitney *U* test.

As the dura mater showed complete vascular regeneration following PTI, whereas the brain contrastingly showed poor vascular recovery, further molecular characterization of these processes was performed in the dura mater and brain. VEGF-A/VEGFR2 signaling has critical roles in angiogenesis and vascular regeneration, whereas VEGF-C/VEGFR3 and angiopoietin-Tie2 signaling play modulatory and facilitating roles in these biological processes^17,18^. Notch signaling, on the other hand, provides counterbalance to these driving forces via Dll4 in certain settings^19,20^. In the normal state, VEGFR2 and Tie2 could be readily detected in both the dura mater and brain BVs (Supplementary Fig. 3a–c, g), whereas Ang2 and Dll4 were hardly detected in either tissue (Supplementary Fig. 3d, e, h, i). At D3 however, when active sprouting angiogenesis occurs, VEGFR2, Ang2, and Dll4 were highly increased, whereas Tie2 was drastically reduced and no VEGFR3, which is restricted to lymphatic vessels, was detected in the ECs of BVs within and surrounding the injury areas of the dura mater (Fig. 2a, Supplementary Fig. 3c–f). In the injured brain, all molecules, excluding VEGFR3, were upregulated in the BVs within and surrounding the injury area (Fig. 2a, Supplementary Fig. 3g–i).

To assess the functional roles of VEGFR2, Tie2, and Notch signaling in vascular regeneration, we deleted *VEGFR2*, *Tie2,* or *Dll4* in ECs in a tamoxifen-dependent manner using *VEGFR2*^i∆EC^, *Tie2*^i∆EC^, or *Dll4*^i∆EC^ mutants, which were generated by crossing *VE-cadherin*-Cre-ER^T2^ mice^21^ with *VEGFR2*^*fl/fl*^ mice^22^, *Tie2* ^*fl/fl*^ mice^23^, or *Dll4*^*fl/fl*^ mice^24^ (Fig. 2b). Cre-ER^T2^-negative but flox/flox-positive littermates were defined as wild-type control mice (control) in each experiment. D7 was chosen as the analyses timepoint, given that dura mater vascular density regenerates to pre-injury levels by 7 days after PTI. The vascular regeneration in the dura mater was drastically compromised by 88% in *VEGFR2*^i∆EC^ mice, whereas it was reduced by 44% in *Tie2*^i∆EC^ mice (Fig. 2c–f) at D7. However, a longer period of healing allowed complete vascular regeneration in *Tie2*^i∆EC^ mice, whereas *VEGFR2*^i∆EC^ mice showed 91% reduction in vascular density in the injured dura mater at D14 (Supplementary Fig. 4a, b), indicating that VEGFR2 signaling is the predominant driver, whereas Tie2 signaling supports vascular regeneration only at the initial stage. Excessive angiogenesis with 43% increase in vascular density occurred with *Dll4* deletion (Fig. 2e, f), implicating the critical role of Notch signaling in terminating angiogenesis when dura mater vascular regeneration is complete. Vascular density in the injured brain was only affected in the *VEGFR2*^i∆EC^ mice, in which 59% decrease was observed (Fig. 2g–j). To confirm the critical dependency of vascular regeneration on VEGFR2 signaling, VEGFR2 blocking antibody DC 101 (40 mg/kg of body weight) or control antibody IgG-Fc (40 mg/kg of body weight) was administered after PTI. Revascularization of avascular areas in the dura mater and brain was markedly suppressed by 74 and 56% with DC 101 treatment (Supplementary Fig. 5a–e), confirming that VEGFR2 signaling is critical for complete vascular regeneration in the injured dura mater. VEGFR2 signaling inhibition also suppressed vascular leakage in the brain by 44% with DC 101 treatment.

**Fig. 3:**
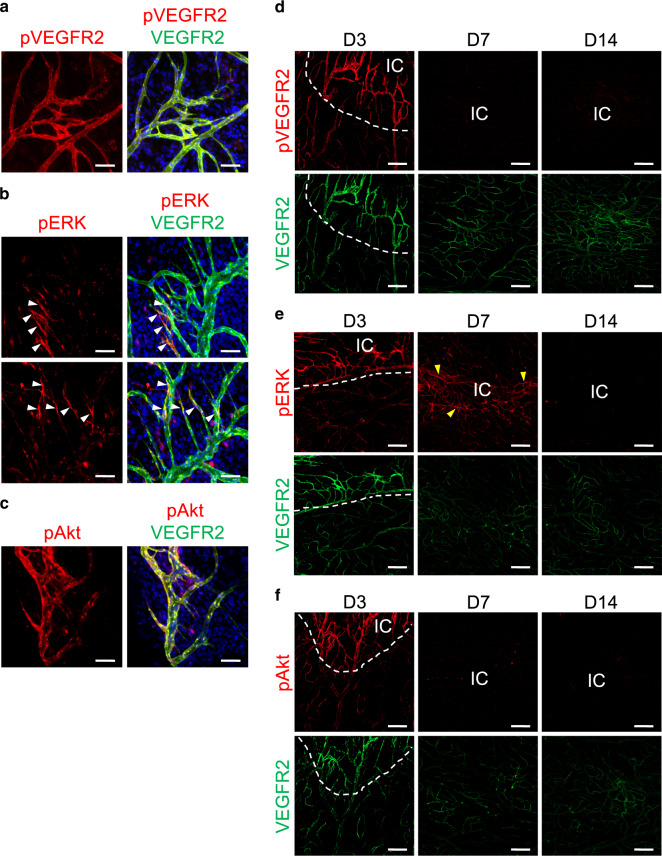
Spatiotemporal changes of pVEGFR2, pERK, and pAkt in VEGFR2+ blood vessels during vascular regeneration in dura mater after PTI. **a**–**c** Representative images of phosphorylated VEGFR2, ERK (white arrowheads), and Akt in regenerating BVs of the dura mater at D3 after PTI in adult mice. Scale bars, 50 µm. Similar findings were observed in four mice from two independent experiments. **d**–**f** Representative images of pVEGFR2, pERK, and pAkt in VEGFR2+BVs of surrounding BVs of putative injury area (white dotted line) or injury core (IC) at D3, D7, and D14 after PTI in adult mice. Similar findings were observed in *n* = 3 mice from two independent experiments. Scale bars, 200 µm. Note that pERK remains elevated at D7 (yellow arrowheads) before returning to basal levels by D14.

High VEGFR2 was well-coupled with pVEGFR2 and pERK in both tissues (Fig. 3a, b, Supplementary Fig. 6a, b) at D3, indicating that vascular regeneration is mainly mediated through activation of VEGFR2-ERK signaling. However, pAkt coupling was far more pronounced in the recovering vessels of the dura mater (Fig. 3c), compared with those of the brain (Supplementary Fig. 6c), and may confer angiogenic advantage to the former. Further temporal analyses of these phosphorylated molecules in the injury area of the dura mater showed pVEGFR2 and pAkt levels returning to basal levels by D7, and pERK by D14, all of which corresponded with the accompanying decrease in VEGFR2 (Fig. 3d–f). Significant increase of VE-PTP, an endothelium-specific vascular endothelial-phosphotyrosine phosphatase^25^, in regenerating vessels of both the dura mater and brain at D3 (Supplementary Fig. 7a–d) suggests that VE-PTP may be involved in such downstream regulation of VEGFR2^26^.

**Fig. 4:**
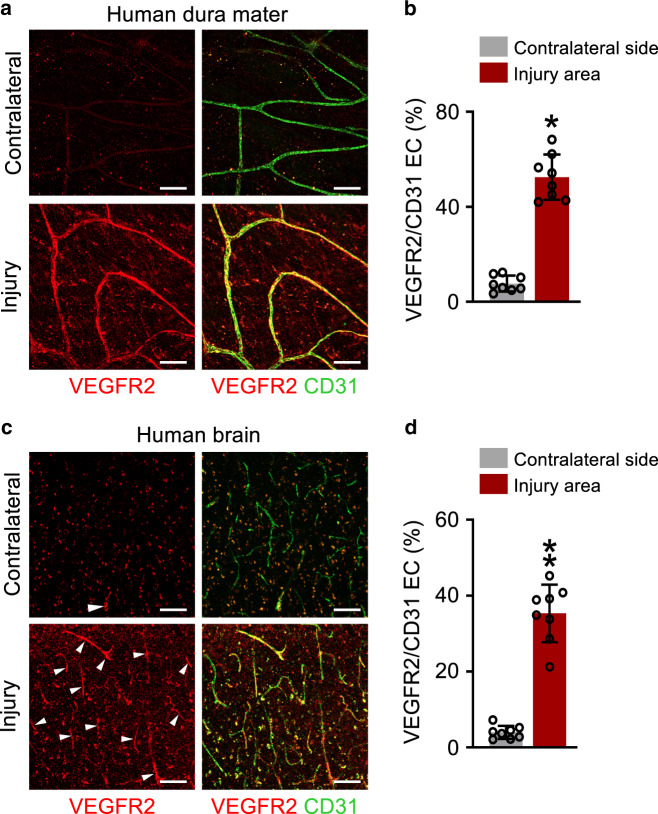
VEGFR2 upregulation in blood vessels of injured dura mater and brain in human patients with traumatic head injury. **a**–**d** Representative images and comparisons of VEGFR2 upregulation in the BVs of injury area and contralateral (CL) side of human dura mater and brain at 24 h post-mortem from patients with HI. White arrowheads indicate VEGFR2 + BVs. Scale bars, 100 µm. Two values were obtained from two areas of each patient sample, *n* = 4 patients from four independent experiments. Vertical bars indicate mean ± SD. **P = 0.0002 versus CL by two-tailed Mann–Whitney *U* test.

Importantly, immunofluorescence analysis of human dura mater and brain cortex autopsy samples obtained from HI patients also showed substantial increase of VEGFR2 in both the dura mater and brain BVs within the injury area when compared with the contralateral side (Fig. 4a–d), underscoring the clinical relevance of increased VEGFR2 in HI.

Therefore, these results implicate key molecular pathways governing vascular regeneration in the dura mater following PTI, with VEGFR2 signaling playing a predominant and critical role.

### PDGFRβ+ cells stabilize vascular regeneration after injury

Pericytes or perivascular cells play diverse roles in angiogenesis, vascular remodeling, regeneration and stabilization^27^. We labeled PDGFRβ+ cells using a PDGFRβ-Cre-ER^T2^-tdTomato reporter mouse model (Supplementary Fig. 8a). Although most PDGFRβ+ cells are tightly associated with CD31+ brain capillaries as pericytes, they are spatially dichotomous in the dura mater, where they are either associated with dural BVs or randomly distributed in the stroma like fibroblasts (Supplementary Fig. 8b). Disorganized accumulation of PDGFRβ+ cells surrounding angiogenic vessels occurred in both the dura mater and brain at D3 (Supplementary Fig. 8c). To address the role of PDGFRβ+ cells in vascular regeneration, PDGFRβ-blocking antibody APB5^28^ or control antibody IgG-Fc was administered after PTI. Although there was no notable difference in the vascular density of the recovered area, the diameter of the recovered vessels was increased by 2.3-fold in the dura mater and brain with APB treatment compared with those with IgG-Fc treatment (Supplementary Fig. 8d, e). Further characterization of NG2+ perivascular cells after APB5 treatment showed significantly decreased NG2+ cell number, as well as poor perivascular coverage by these cells (Supplementary Fig. 8f, g). Thus, PDGFRβ+ cells play a stabilizing role in the overall structure of the regenerative BVs in the dura mater and brain upon injury.

### Diverse macrophage behaviors in meninges and brain after PTI

It has recently been demonstrated that F4/80+, LYVE1+, and CD206+ macrophages are adjacently aligned with capillaries of the dura mater, and play surveillance roles as the secondary interface between the systemic circulation and the CNS^10,29^. As LYVE1+ macrophages have been reported to contribute to angiogenesis and BV homeostasis in various tissues^30–32^, we examined the spatiotemporal distribution of LYVE1+ macrophages after PTI. Distribution of LYVE1+/F4/80+ macrophages was markedly reduced in the dura mater injury core during vascular regeneration, but they were highly distributed with activated shapes at the injury margin at D3 (Supplementary Fig. 9a–c). In contrast, single F4/80+ macrophages were profoundly accumulated within the injury core with activated shapes at D3 (Supplementary Fig. 9a–c). Beneath the dura mater, distribution of LYVE1+/F4/80+ macrophages was confined to the pia mater, whereas single F4/80+ macrophages were heavily accumulated in the injury core of brain at D3 and D7 (Supplementary Fig. 9d, e). Given that residential dura mater macrophages and brain microglia are distinct from bone marrow-derived circulating macrophages, we investigated the origin of the activated macrophages in the injury area following PTI. Analyses of WT:actin-GFP parabiosis recipient mice showed complete absence of GFP-expressing macrophages in the injured dura mater, whereas a significant proportion of macrophages in the injured brain expressed GFP (Supplementary Fig. 9f–h). These results indicate that activation and accumulation of dura mater macrophages occur internally, whereas these processes occur in the brain with substantial contribution from circulating monocytes/macrophages.

### Macrophages play minor role in dural vascular regeneration

**Fig. 5:**
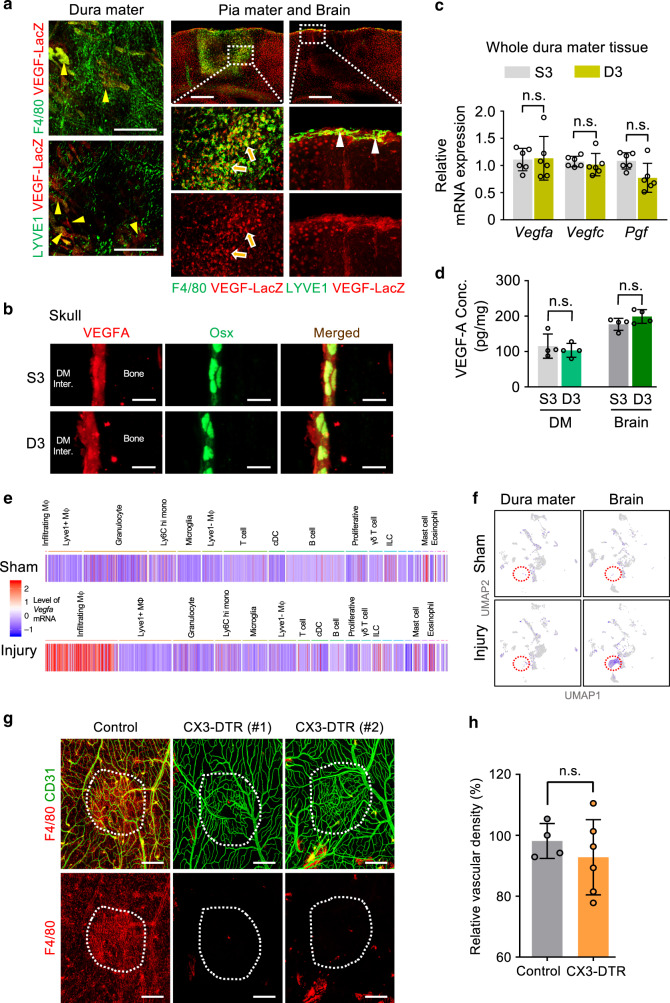
Dura mater macrophages play minor role in meningeal vascular regeneration. **a** Representative images of VEGF-A expression by detection of β-galactosidase activity in VEGF-LacZ mice at D3. High expression of VEGF-A is detected in neighboring calvarial bone (yellow arrowheads), F4/80+ macrophages (yellow arrows) in brain, and LYVE1+ macrophages (white arrowheads) in pia mater. Similar findings were observed in *n* = 3 mice from two independent experiments. Scale bars, 500 µm. **b** Representative images of VEGF-A in Osterix+ (Osx+) osteogenic progenitor cells/osteoblasts along the endosteal surface of calvarial bone of adult mice at D3 after PTI. No difference in VEGF-A between S3 and D3 is shown. Similar findings were observed in *n* _= 3 mice from two independent experiments. DM Inter., dura mater interface. Scale bars, 10 µm. **c**, **d** Relative mRNA expressions of *Vegfa, Vegfc,* and *Pgf* in whole dura mater tissue and VEGF-A protein concentrations in the dura mater (DM) and brain at D3. Each dot indicates a value obtained from one mouse and *n* = 6 **c**, *n* = 4 **d** mice/group from three independent experiments. Vertical bars indicate mean ± SD. n.s., not significant versus each S3 by two-tailed Mann–Whitney *U* test. **e** Heatmap of *Vegfa* expression based on single-cell RNAseq analysis of CD45+ hematopoietic cells isolated from dura mater and brain at D3. Emergence of infiltrating macrophages with high *Vegfa* expression is observed after PTI. **f** Feature plots showing *Vegfa* expression in various CD45+ hematopoietic cell clusters at D3. Note emergence of infiltrating macrophages showing high *Vegfa* expression only in brain after PTI (red dotted circle). **g**, **h** Representative images and comparisons of vascular regeneration in the dura mater between control mice and CX3CR1-DTR mice (two cases #1 and #2 are shown) at D7. White dotted-lined circles indicate putative injury areas. Scale bars, 500 µm. Each dot indicates a mean value obtained from one mouse and *n* = 4 (S3), *n* = 6 (D3) mice/group from two independent experiments. Vertical bars indicate mean ± SD. n.s., not significant versus control by two-tailed Mann–Whitney *U* test.

Activated macrophages are known to be major suppliers of VEGF-A in the context of tissue injury^33,34^. To clarify the predominant source of VEGF-A during vascular regeneration, we examined the expression and production of VEGF-A using a VEGF-LacZ reporter mouse model. VEGF-A was not upregulated in both LYVE1+/F4/80+ and single F4/80+ macrophages in the dura mater at D3 (Fig. 5a). In comparison, VEGF-A was highly upregulated in single F4/80+ macrophages in the injury core of brain and in LYVE1+/F4/80+ macrophages in the pia mater at D3 (Fig. 5a). VEGF-A expression was predominantly high in Osterix+ osteoprogenitors/osteoblasts in the calvarial bone (Fig. 5a, b) and in the normal portions of neurons in the brain (Fig. 5a). RT-PCR analysis of the dura mater revealed no significant differences of mRNA expression of *Vegfa*, *Vegfc*, and *Pgf* between sham-operated day 3 (S3) and D3 (Fig. 5c). Moreover, enzyme-linked immunosorbent assay (ELISA) revealed no significant difference of VEGF-A_164_ in the dura mater and brain between S3 and D3 (Fig. 5d). Single-cell RNAseq analyses of fluorescence-activated cell sorting (FACS)-sorted CD45+ hematopoietic cells (Supplementary Fig. 10a, b) showed sparse *Vegfa* expression in various immune cells, such as in granulocytes, mast cells, innate lymphoid cells, and eosinophils of the dura mater and brain in the sham condition (Fig. 5e). *Vegfa* was largely absent in macrophages of both tissues and in brain-resident microglia populations. Upon injury, a new macrophage population expressing strong *Vegfa* levels emerged within the injured brain, whereas no *Vegfa* expression changes could be detected within hematopoietic cells of the dura mater following PTI (Fig. 5e, f).

Finally, to evaluate the potential contribution of these macrophages to vascular regeneration, we genetically depleted monocytes/macrophages using a *Cx3cr1*^DTR^ mutant mouse model. Systemic administration of diphtheria toxin ablated macrophages in most tissues by 85−98% and caused 64% and 46% reductions, respectively, in vascular regeneration in the injured pia mater and brain, respectively (Supplementary Fig. 11a–f), but impact on dura mater vascular regeneration was minimal (Fig. 5g, h). These data indicate that infiltrating macrophages expressing high VEGF-A in the pia mater and brain are required for vascular regeneration, whereas accumulated macrophages in the dura mater play minor facilitating roles in vascular regeneration.

### Inflammatory role of dura mater macrophages following PTI

As high VEGF-A expression was found only in activated macrophages of the injured brain (Supplementary Fig. 12a), we sought to explore other angiogenic factors expressed by dura mater macrophages. Single-cell RNAseq analyses of CD45+ hematopoietic cells found in the dura mater and brain showed a distinct *Lyve1*+ sub-cluster of macrophages (Supplementary Fig. 10a, b). Further comparative analyses between *Lyve1*+ and *Lyve1*− macrophages within the dura mater revealed that the expression of multiple MHC class II molecules was enriched in the latter (Supplementary Fig. 12b), suggesting the specialized immune-surveillance role of these cells in dura mater protection against infection. Following injury, these dura mater-resident *Adgre1*+ macrophages showed elevated expression of pro-inflammatory molecules, such as *Ccl2*, *Ccl7*, *Ccl8*, *Ccl9,* and *Cxcl2* (Supplementary Fig. 12d). Significant changes in pro-angiogenic molecules could not be detected in the *Lyve1*+ or *Lyve1*−macrophages of the injured dura mater. Brain-infiltrating macrophages, on the other hand, had increased expression of a wide range of angiogenic and anti-inflammatory molecules, such as *Vegfa*, *Lgals1* & *3*, and *Iqgap1* (Supplementary Fig. 12c, e). Interestingly, anti-angiogenic molecules, such as *Thbs1*, *Runx3*, *Tmsb10*, *Tgfbi,* and *Pgk1*, were also upregulated, suggesting another potential explanation for poor vascular regeneration in the injured brain. Therefore, these results confirm the absence of increased angiogenic contribution from dura mater macrophages, which are quite unique in molecular nature, or from any other hematopoietic cell within the injured dura mater.

### VEGFR2 signaling is required for dural vascular homeostasis

**Fig. 6:**
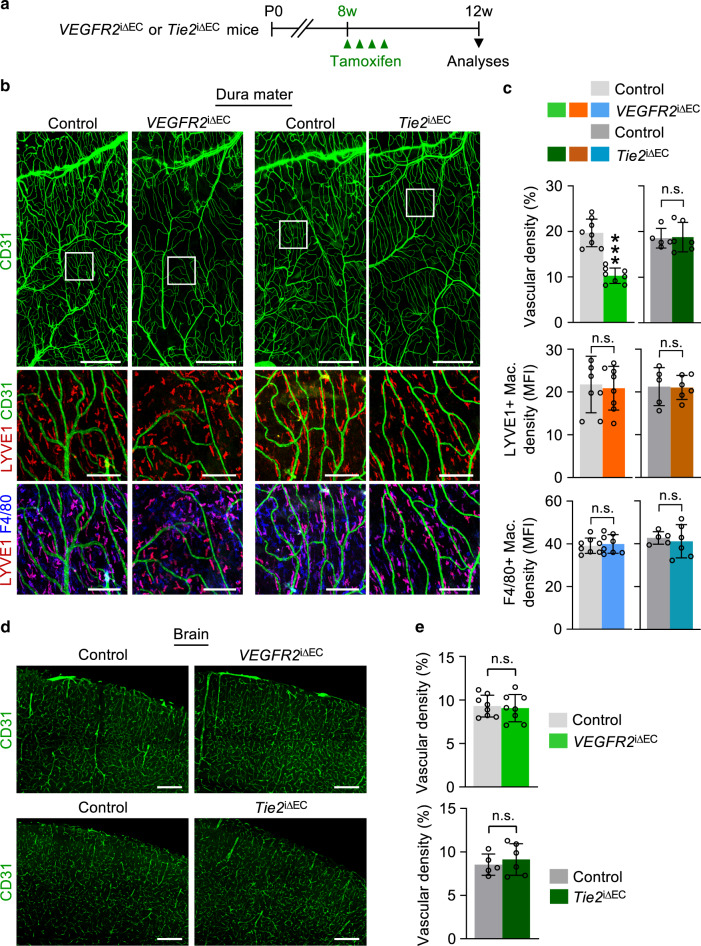
VEGFR2, but not Tie2, signaling is required for maintaining dural vascular homeostasis. **a** Diagram depicting generation of *VEGFR2*^iΔEC^ or *Tie2*^iΔEC^ mice and EC-specific deletion of *VEGFR2* or *Tie2* at 8 weeks old of age and their analyses 4 weeks later. **b** Representative images of CD31+BVs (upper panels) and distributions of LYVE1+ (middle and lower panels) and F4/80+ macrophages (lower panels) in dura mater of control, *VEGFR2*^iΔEC^ and *Tie2*^iΔEC^ mice. Scale bars, 1 mm. Each white-lined box is magnified in middle and lower panels. Scale bars, 200 µm. **c** Comparisons of densities of CD31+BVs, LYVE1+macrophages and F4/80+ macrophages in control and *VEGFR2*^iΔEC^ or *Tie2*^iΔEC^ mice. Each dot indicates a value from one mouse and *n* = 5 (*Tie2* control), *n* = 6 (*Tie2*^iΔEC^), *n* = 8 (all other groups) mice/group from three independent experiments. Vertical bars indicate mean ± SD. n.s., not significant or ***P < 0.001 versus control by two-tailed Mann–Whitney *U* test. MFI, mean fluorescence intensity. **d**, **e** Representative images of CD31+BVs in brain of control, *VEGFR2*^iΔEC^, and *Tie2*^iΔEC^ mice. Scale bars, 200 µm. Comparisons of vascular density between control and *VEGFR2*^iΔEC^ or *Tie2*^iΔEC^ mice. Each dot indicates a value from one mouse and *n* = 5 (*Tie2* control), *n* = 6 (*Tie2*^iΔEC^), *n* = 8 (all other groups) mice/group from three independent experiments. Vertical bars indicate mean ± SD. n.s., not significant versus control by two-tailed Mann–Whitney *U* test.

Our observation of robust VEGFR2 and Tie2 expression in dural BVs led us to explore the roles of VEGFR2 and Tie2 signaling in adult vascular homeostasis by examining the phenotypes of BVs and perivascular macrophages in the *VEGFR2*^i∆EC^ and *Tie2*^i∆EC^ mice (Fig. 6a). Compared with those of control mice, diameters of capillaries and venules were ∼50% thinner and vascular density was reduced by 47% in *VEGFR2*^i∆EC^ mice but remained similar in *Tie2*^i∆EC^ mice (Fig. 6b, c). Nevertheless, no differences were found in vascular density of the brain, and distribution, alignment and density of LYVE1+/F4/80+ and single F4/80+ perivascular macrophages among control, *VEGFR2*^i∆EC^, and *Tie2*^i∆EC^ mice (Fig. 6b–e). These findings indicate that VEGFR2, but not Tie2, signaling is required to maintain dural vascular homeostasis.

### Dura mater ECs intrinsically possess angiogenic potential

**Fig. 7:**
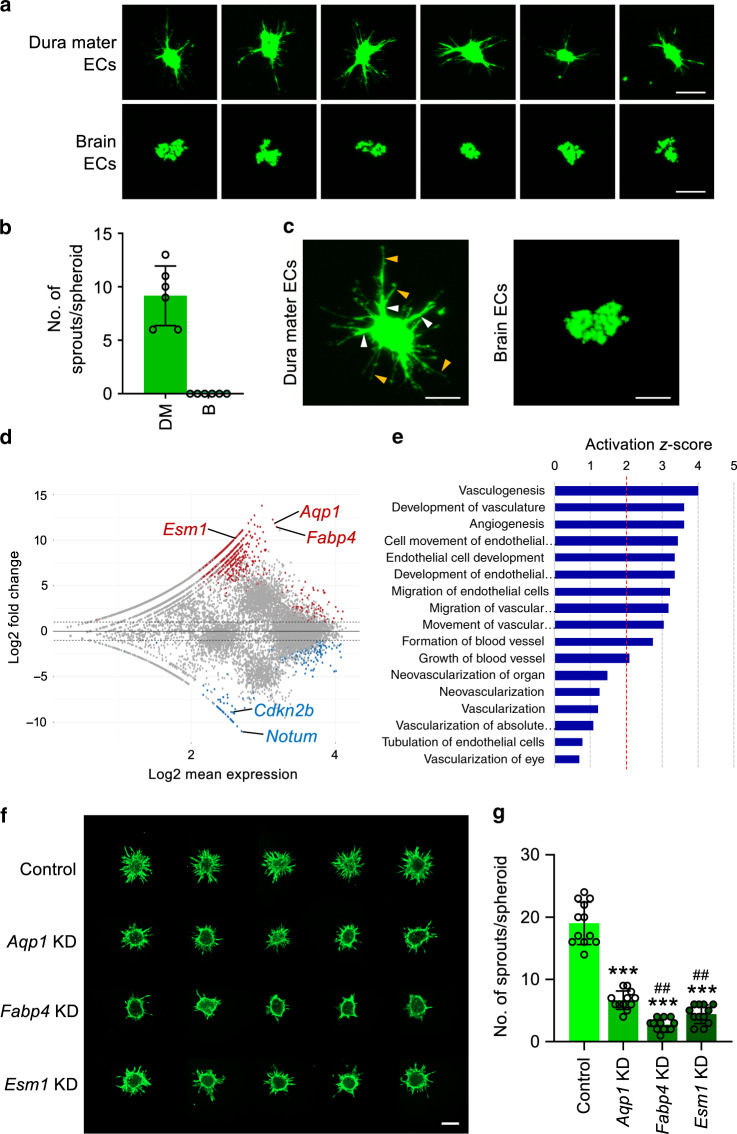
Dura mater ECs are superior to brain ECs in sprouting activity and show high expression of angiogenic molecules. **a**, **b** Images and comparisons of the number of sprouts (length, >50 μm) of EC spheroids derived from FACS-sorted primary ECs of dura mater (DM) and brain (B) at 12 hr after incubation in collagen type I gel. Each dot indicates number of sprouts per spheroid. Each *n* = 6 from two independent experiments. Vertical bars indicate mean ± SD. *P* = 0.0022 versus brain ECs by two-tailed Mann–Whitney *U* test. Scale bars, 100 μm. **c** Highly magnified images of the left two panels in **a** showing robust sprouting (white arrowheads) and filopodia (yellow arrowheads) in EC spheroid from DM, whereas no sprouting and filopodia are seen in EC spheroid from B. Scale bars, 50 μm. **d** MA plot of significantly high-(1020 genes, red) and low (323 genes, blue)-expressing genes in dura mater ECs compared with brain ECs. Dotted horizontal lines demarcate twofold change between dura mater ECs and brain ECs. **e** IPA of 1343 differentially expressed genes in dura mater ECs compared with brain ECs. Ranked according to activation *z* score within Cardiovascular System Development and Function category. Vertical red-dotted line marks activation *z* score threshold. *P* < 0.001 for all pathways by right-tailed Fisher’s Exact Test with Benjamini–Hochberg correction for multiple testing. **f**, **g** Representative images and comparisons of the number of sprouts (length, >100 μm) from HUVEC spheroids at 24 h after incubation in collagen type I gel. siRNA-mediated KD was induced before spheroid formation. Scale bars, 200 μm. Each dot indicates number of sprouts per spheroid. Each *n* = 12 from two independent experiments. Vertical bars indicate mean ± SD. ***P < 0.001 versus control, ^##^*P* < 0.01 versus *Aqp1* KD by Kruskal–Wallis test.

To assess EC-intrinsic advantages to vascular regeneration, an ex vivo sprouting assay^35^ was performed using FACS-sorted dura mater or brain ECs. Although robust sprouting and filopodia were observed in dural EC spheroids, almost no sprouting and filopodia were detected in brain EC spheroids (Fig. 7a–c), confirming that dura mater ECs have a more vigorous intrinsic angiogenic potential than brain ECs.

We then performed RNAseq analysis to further elucidate fundamental molecular differences between these tissue-specific ECs. A majority of 1020 genes from 1343 differentially expressed genes (DEGs) between dura mater and brain ECs were upregulated in the former (Fig. 7d). Ingenuity Pathway Analysis (IPA) analysis of these changes showed strong enrichment of vascular development and angiogenesis pathways (Fig. 7e, Supplementary Fig. 13a, b). Interestingly, integrin signaling and integrin-linked kinase signaling showed high enrichment in the dura mater ECs (Supplementary Fig. 13c), suggesting that certain molecules within these pathways may contribute to the angiogenic state of the dura mater by cooperating with VEGFR2 as previously described^17^. siRNA-mediated knockdown of several candidates found highly enriched in dura mater ECs (*Aqp1*, *Fabp4,* and *Esm1*) significantly impaired sprouting angiogenesis by 65%, 84%, and 76%, respectively (Fig. 7f, g).

### Complete regeneration of dura mater vessel basement membrane

**Fig. 8:**
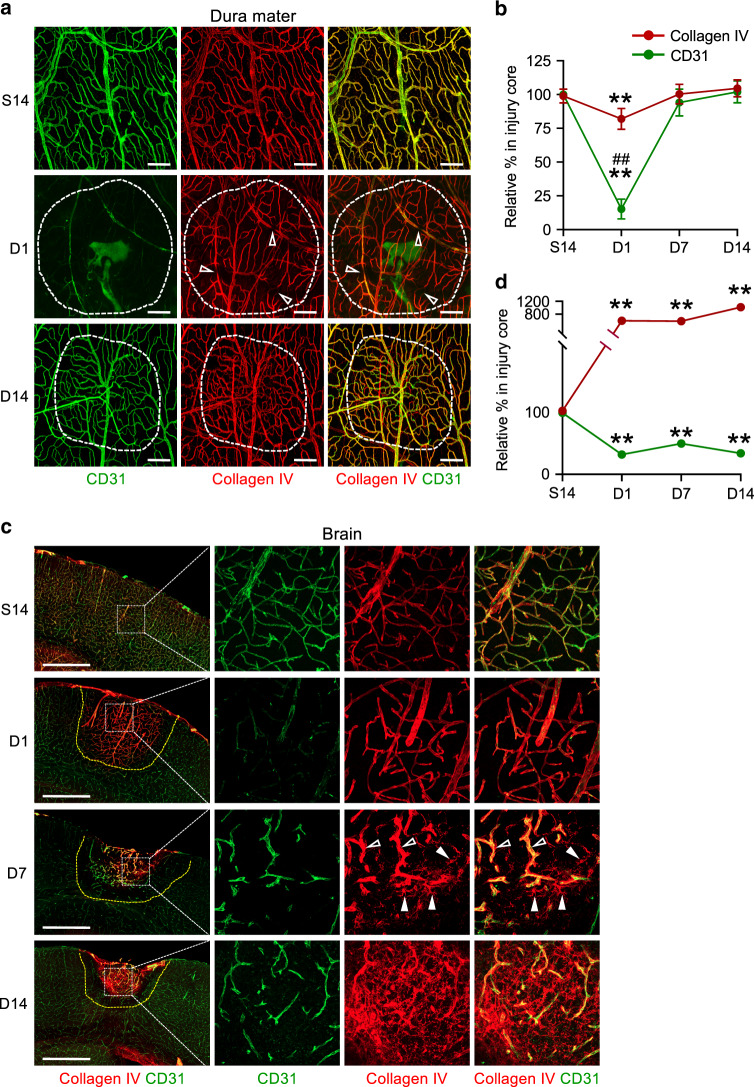
Rapid recovery of BV basement membrane in the injured dura mater but not in the injured brain after PTI. **a**, **b** Representative images and comparisons of distributions of collagen IV along CD31+BVs in putative injury area (white dotted-lined circle) at indicated days in the dura mater of adult mice after PTI. Empty arrowheads indicate broken alignment of collagen IV. Scale bars, 200 µm. *n* = 4 mice/group from two independent experiments. Mean fluorescence intensity (MFI) of collagen IV per MFI of CD31 in the putative injury area at S14 is regarded as 100%. Dots and error bars indicate mean ± SD. **P < 0.01 versus S14 and ^##^*P* < 0.01 versus D1 of collagen IV by Kruskal–Wallis test. **c**, **d** Representative images and comparisons of distributions of collagen IV and CD31+BVs in putative injury area (white dotted line) at indicated days in the brain of adult mice after PTI. Scale bars, 500 µm. Each indicated box area is magnified and arrayed in the right panels. Empty arrowheads indicate increased collagen IV in perivascular areas, whereas white arrowheads indicate collagen IV accumulation in avascular areas. *n* = 4 mice/group from two independent experiments. MFI of collagen IV or MFI of CD31 in the putative injury area at S14 is regarded as 100%. Dots and error bars indicate mean ± SD. **P < 0.01 versus S14 by Kruskal–Wallis test.

We further characterized vascular responses to PTI in the dura mater and brain to identify additional tissue-specific differences. Of note, collagen IV+ basement membrane (BM) along the dural vessels was relatively well preserved, similar to tumor vessel regression^36^, following injury, whereas CD31+ECs were markedly damaged at D1 (Fig. 8a, b). At D14, by which vascular function is fully restored to pre-injury conditions, the alignment of collagen IV + BM and shape of the regenerated BVs fully recovered, looking like pre-injury normal BVs, in the dura mater (Fig. 8a, b). In contrast, collagen IV along the brain vessels was highly increased at D1 and continued to accumulate, irrespective of vascular patterning, within the injury core until D14 (Fig. 8c, d), highlighting the superior functional regeneration, with extrinsic BM support, of dura mater BVs following PTI.

### Vascular regeneration is required for dural tissue recovery

**Fig. 9:**
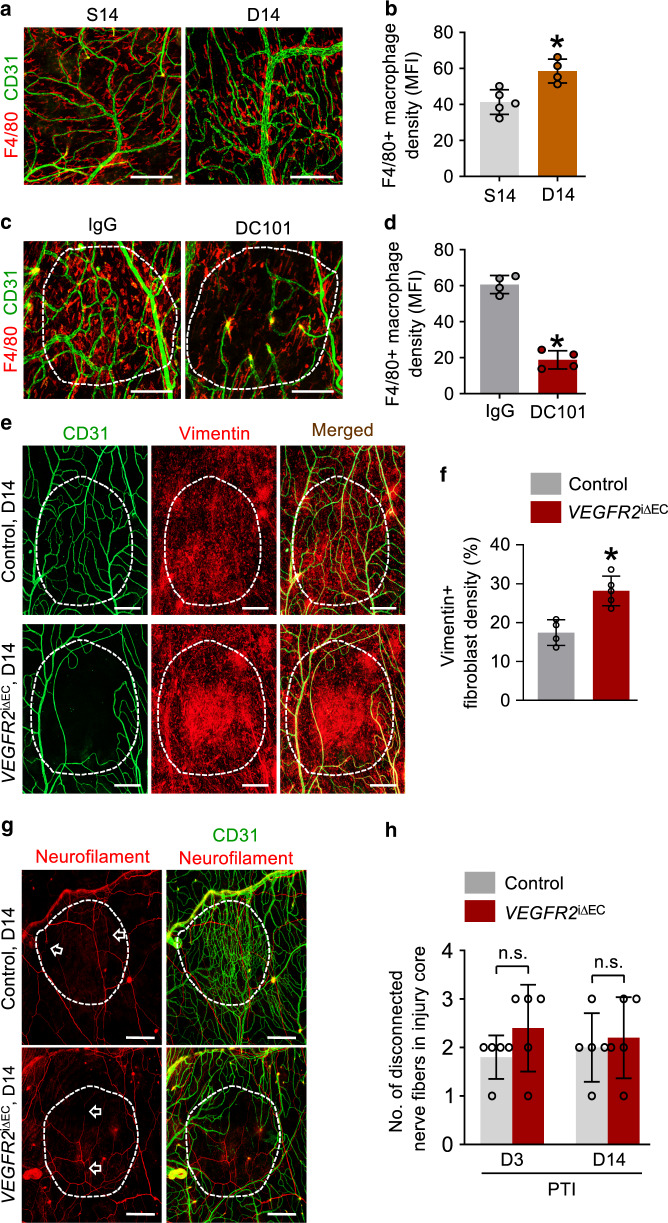
Vascular regeneration is required for recovery of perivascular macrophages and normalization of fibroblasts in dura mater after PTI. **a**, **b** Representative images and comparisons of the distribution of F4/80+ macrophages in the putative injury area (white dotted-lined circle) of dura mater at S14 and D14 in adult mice. Scale bars, 200 µm. Each dot indicates a mean value obtained from one mouse and *n* = 5 (S14), *n* = 4 (D14) mice/group from two independent experiments. Vertical bars indicate mean ± SD. *P = 0.0159 versus S14 by two-tailed Mann–Whitney *U* test. MFI, mean fluorescence intensity. **c**, **d** Representative images and comparisons of the distribution of F4/80+ macrophages in the putative injury area (white dotted-lined circle) of dura mater at D14 in adult mice that were treated with IgG-Fc or DC101 (daily i.p. injections of 40 mg/kg of body weight for 6 days). Note substantial inhibition of F4/80+ macrophage distribution in the injury area with DC101 treatment. Scale bars, 200 µm. Each dot indicates a mean value obtained from one mouse and *n* = 4 mice/group from two independent experiments. Vertical bars indicate mean ± SD. *P = 0.0286 versus IgG-Fc by two-tailed Mann–Whitney *U* test. MFI, mean fluorescence intensity. **e**, **f** Representative images and comparisons of the distribution of vimentin+ fibroblasts in the putative injury area (white dotted-lined circle) of dura mater in control and *VEGFR2*^iΔEC^ mice at D14. Scale bars, 200 µm. Each dot indicates a mean value obtained from one mouse and *n* = 4 (control), *n* = 5 (*VEGFR2*^iΔEC^) mice/group from two independent experiments. Vertical bars indicate mean ± SD. *P = 0.0159 versus control by two-tailed Mann–Whitney *U* test. **g**, **h** Representative images and comparisons of the number of broken neurofilament+nerve fibers (arrows) in the putative injury area (white dotted-lined circle) of dura mater in control and *VEGFR2*^iΔEC^ mice at D3 and D14. Scale bars, 200 µm. Each dot indicates a mean value obtained from one mouse and *n* = 5 mice/group from three independent experiments. Vertical bars indicate mean ± SD. n.s., not significant by two-tailed Mann–Whitney *U* test.

Considering the role of vascular regeneration in dura mater function, we investigated the consequences of incomplete vascular regeneration in the dura mater. Vascular regeneration inhibition with prolonged DC101 treatment for 2 weeks following PTI showed a substantial reduction in dura mater-resident F4/80+ macrophages in the avascular area (Fig. 9a–d). We also examined the extent of fibrosis within the avascular area using the fibroblast marker vimentin. After prolonged inhibition of vascular regeneration following PTI in EC-specific *VEGFR2* knockout mice, density of vimentin+ fibroblast was increased by ∼50% in *VEGFR2*^i∆EC^ mice compared with control mice at D14 (Fig. 9e, f), implying that complete dura mater vascular regeneration is required to prevent excessive fibrosis at the injury site. Similarly, inhibition of VEGFR2-mediated vascular regeneration following an ear skin wound also led to the increase of fibrotic tissue surrounding the injury core at D14 (Supplementary Fig. 14a, b). However, no difference in the number of broken nerve fibers was observed at D14 in the dura mater between control and *VEGFR2*^i∆EC^ mice (Fig. 9g, h), suggesting that vascular regeneration and peripheral nerve regeneration are not coupled in this context. Therefore, these results implicate efficient vascular regeneration as a critical mediator in the restoration of immune homeostasis and wound healing of the injured area following HI.

## Discussion

Our study identifies key pathways of vascular regeneration in the dura mater following HI. We implicate VEGFR2 signaling as the predominant driver for dura mater vascular homeostasis and regeneration, with multiple intrinsic/extrinsic factors creating a primed state for rapid vascular regeneration.

Various impact injury models^37–39^ better simulate direct tissue damage from head trauma. However, the PTI model employed in this study is most useful for the side-by-side comparison of the meninges and the brain parenchyma following vascular injury, because it allows minimally invasive, deep-penetrating, consistent, and direct injury of BVs, with most tissue damage hallmarks associated with head trauma.

A previous study attributes dura mater vascular regeneration to macrophage-supplied MMP-2, which facilitates vascular regeneration by breaking down fibrin deposits accumulated within the injury core^14^. Our results do not show changes in VEGF-A levels but indicate that endothelial VEGFR2 signaling is upregulated, both in human HI patients and mice, and drives dura mater vascular regeneration. Unlike minimal suppression of vascular regeneration with macrophage depletion, we observed complete inhibition of vascular regeneration with EC-specific deletion or antibody blockade of VEGFR2. In contrast to low transcription levels, substantial amounts of VEGF-A protein in the dura mater suggest its supply from neighboring tissue, such as calvarial bone or neurons.

Although our study demonstrates that both the meninges and brain vessels rely on VEGFR2 for their differential vascular regeneration following PTI, molecular profiling of dura mater vs brain ECs based on RNAseq suggests that other specialized pathways may confer angiogenic and vascular regenerative advantages upon dura mater ECs and warrants further investigation. Also, how such dura mater EC-specific molecules contribute to suppressing vascular leakage, despite lacking a blood–brain barrier present in the brain^9^, remains to be explored.

Although the functional significance of meningeal lymphatics has recently received the spotlight^40–42^, the role of meningeal BVs has been rather unclear. Our study demonstrates the critical role dura mater BVs play in the functional regeneration of the dura mater tissue, with respect to its immunological function and stromal integrity. Although the dura mater vascular bed is separate from the pia mater and brain interconnected beds^8,9^ and therefore does not inflict direct vascular damage to the latter if injured, how the functional recovery of the dura mater potentially affects brain function also requires further investigation. While the debate on employing anti-VEGF therapy to manage cerebral edema in stroke and HI patients continues^12,43–46^, our results suggest that targeting this angiogenic pathway in HI patients may impede dura mater recovery and compromise its primary functions.

Therefore, our study provides an important molecular framework for dura mater vascular biology by identifying several key and organotypic EC molecules in dura mater BV maintenance and regeneration following injury, and establishes the functional role of these tissue-specific BVs in dura mater regeneration following HI.

## Methods

### Mice

Specific pathogen-free (SPF) C57BL/6 J were purchased from DBL Co., Ltd. (Republic of Korea). Actin-GFP, *Cx3cr1*^DTR^, and R26-tdTomato mice were purchased from Jackson Laboratory. *VE-cadherin*-Cre-ER^T2^ ^21^, *VEGF-A*^+/LacZ^ ^47^ and *VEGFR2*^[fl/fl 22^, *Tie2*^fl/fl^ ^23^, *Dll4*^fl/fl^ ^24^, and PDGFRβ-Cre-ER^T2^ ^48^ mice were transferred, established, and bred in SPF animal facilities at KAIST. All mice were maintained in the C57BL/6 background, housed under 12 hr light/12 hr dark cycle, temperatures of 22 ± 2 °C with 50 ± 10% humidity, and fed with free access to a standard diet (PMI LabDiet) and water. In order to induce Cre activity in the Cre-ER^T2^ mice, 2 mg of tamoxifen (Sigma-Aldrich) dissolved in corn oil (Sigma-Aldrich) was injected intra-peritoneally (i.p.) at indicated time points for each experiment. Cre-ER^T2^ negative but flox/flox-positive mice among the littermates were defined as control mice for each experiment. Cre-mediated deletion efficiencies in both dura mater (84–88%) and brain (81–85%) were validated by histological analyses of the targeted proteins (Supplementary Fig. 15).

Mice were anesthetized with i.p. injection of a combination of anesthetics (80 mg/kg ketamine and 12 mg/kg of xylazine) before any procedures or being sacrificed. Animal care and experimental procedures were performed under the approval from the Institutional Animal Care and Use Committee of KAIST (no. KA2018-42). All procedures and animal handlings were performed following the ethical guidelines for animal studies.

### Photothrombotic injury

Mice at 8–10 weeks of age were anaesthetized with ketamine/xylazine immediately prior to surgery. Head fur was removed with pet hair clippers (Himax) and the incision site was sterilized with 70% ethanol. A surgical skin flap was created to expose the calvarium before the animal was mounted on a custom-built fast-scanning confocal microscope used for intravital imaging^49^. White light measured at 3 mW was aimed at the targeted injury site between the coronal and lambdoid sutures of the mouse calvarium and 100 μl of Rose Bengal (15 mg/ml, Sigma-Aldrich) was injected into the retro-orbital vein. After 3 min of white light exposure to the targeted injury site, the surgical skin flap was sutured with a 5–0 (one metric) non-absorbable black braided silk suture (Ailee). Post-surgical care was performed under a heating lamp until mice showed complete recovery from anesthesia.

### Histological analyses

After anesthesia, transcardial perfusion was performed into the mice with phosphate-buffered saline (PBS) followed by 4% paraformaldehyde (PFA, Merck). The dura mater-attached skull and brain of the PTI areas were harvested and the samples were then post-fixed at 4 °C in 4% PFA for 2 h for the dura mater and overnight for brain samples. Dura mater and pia mater samples were whole-mount stained. Brain samples were sectioned at 80 µm thickness using a vibrating microtome (Leica Biosystems) prior to immunostaining. Samples were washed with PBS several times before blocking. After blocking with 5% goat or donkey serum (Jackson ImmunoResearch) in 0.3% Triton-X 100 in PBS (PBST) for 1 h, samples were incubated with the indicated primary antibodies diluted in the blocking solution at 4 °C overnight. After several washes with PBST, the samples were incubated at 4 °C overnight with the indicated fluorochrome-conjugated secondary antibodies diluted in the blocking buffer. The samples were washed with PBST and nuclei were stained with 4′,6-diamidino-2-phenylindole (DAPI) (Invitrogen). After washing with PBS, samples were mounted with Vecta-shield (Vector Laboratories). Images were obtained using Zeiss LSM 800 or LSM 880 confocal microscope (Carl Zeiss). The following primary antibodies were used in the immunostaining of mouse samples: anti-CD31 (hamster monoclonal, MAB1398Z, Merck), anti-pVEGFR2 (Tyr1175, rabbit monoclonal, 2478, Cell Signaling Technology), anti-pERK (Thr202/Tyr204, rabbit monoclonal, 4370, Cell Signaling Technology), anti-pAkt (Ser473, rabbit monoclonal, 4060, Cell Signaling Technology), anti-VE-PTP (rabbit polyclonal, generously provided by Dietmar Vestweber), anti-VEGFR2 (goat polyclonal, AF644, R&D), anti-VEGFR3 (goat polyclonal, AF743, R&D), anti-Tie2 (goat polyclonal, AF762, R&D), anti-Dll4 (goat polyclonal, AF1389, R&D), anti-Ang2^50^, anti-NG2 (rabbit polyclonal, AB5320, Merck), anti-F4/80 (rat monoclonal, MCA497, Bio-Rad), anti-LYVE-1 (rabbit polyclonal, 11-034, Angiobio), anti-VEGF_164_ (goat polyclonal, AF-493-NA, R&D), anti-Osterix (rabbit polyclonal, ab22552, Abcam), anti-collagen type IV (rabbit polyclonal, ab6586, Abcam), anti-vimentin (chicken polyclonal, AB5733, Merck) and anti-neurofilament heavy polypeptide (rabbit polyclonal, ab8135, Abcam). The following primary antibodies were used in the immunostaining of human samples: anti-VEGFR2 (goat polyclonal, AF357, R&D) and anti-CD31 (rabbit polyclonal, ab28364, Abcam). Alexa Fluor 488-, Alexa Fluor 594-, Alexa Fluor 647-conjugated secondary antibodies were purchased from Jackson ImmunoResearch. All the antibodies used in our study were validated for the species and applications. β-gal staining of *VEGF-A*^+/LacZ^ samples was performed as previously described^51^. In brief, samples were washed and permeabilized with staining buffer (2 mM MgCl_2_, 0.01% sodium deoxycholate, and 0.02% NP-40 in PBS) and stained for 4 h in 1 mg/ml X-gal in staining buffer supplemented with 5 mM potassium ferricyanide and 5 mM potassium ferrocyanide. Samples were washed with PBS several times before immunostaining.

### Morphometric analysis

Morphometric measurements were performed using ImageJ software (NIH) or ZEN 2012 software (Carl Zeiss). To determine vascular density and distribution of collagen IV in the mice with PTI or sham operation, MFI of CD31+ or collagen IV+ was measured in dura mater (2.5 mm^2^), pia mater (4.0 mm^2^), and brain (0.36 mm^2^) of the injury or putative injury areas. To determine vascular density and distribution of collagen IV in the genetically modified mice, MFI of CD31+ or collagen IV was measured in dura mater (3.6 mm^2^), pia mater (5.0 mm^2^), and brain (1.0 mm^2^). To measure density of LYVE1+ or F4/80+ macrophages in the dura mater of genetically modified mice, each MFI was measured in area (0.36 mm^2^) of three regions per sample. To measure density of LYVE1+ or F4/80+ macrophages in the mice with PTI or sham operation, each MFI was measured in injured area of dura mater (2.5 mm^2^), pia mater (4.0 mm^2^), and brain (0.36 mm^2^). To measure VE-PTP density in VEGFR2+ BVs, MFI of VE-PTP was measured in the dura mater (0.36 mm^2^) and brain (0.36 mm^2^). To measure density of perivascular cells in the dura mater, MFI of NG2+ cells was measured in the injury area (0.36 mm^2^). To determine density of fibroblasts, MFI of vimentin+ cells was measured in dura mater (1.2 mm^2^) and ear skin (0.20 mm^2^). To determine vascular density in the ear skin, MFI of CD31 was measured in circumferential region (0.10 mm^2^) of punch hole injury. To determine EB leakage, IFI of EB was measured in injured area of dura mater (3.6 mm^2^), pia mater (4.0 mm^2^), and brain (1.0 mm^2^). To measure VEGFR2 in the BVs of human dura mater and brain, each MFI of VEGFR2+ or CD31+ was measured in the indicated area of dura mater (0.36 mm^2^) and brain (0.36 mm^2^).

### EdU incorporation assay for proliferating ECs

To detect proliferating ECs, 5 mg of 5-ethynyl-2ʼ-deoxyuridine (EdU, A10044, Invitrogen) was dissolved in 1 ml of Milli-Q water as a stock solution. Then, 200 μl of the stock solution per mice was injected i.p. every day, starting from 1 day prior to PTI and for 4 days before analysis. Tissues were isolated and processed as described above. EdU-incorporated cells were detected with the Click-iT EdU Alexa Fluor 488 Assay Kit (Invitrogen) according to the manufacturer’s protocol.

### Lectin perfusion assay

Mice were anaesthetized immediately prior to retro-orbital injection of fluorescein isothiocyanate (FITC)-conjugated lectin (Sigma-Aldrich, 100 µg/100 µl). Animals were killed 30 min after injection, processed and immunostained as described above. FITC was detected in the 488 nm channel but pseudo-colored to red using ZEN software (Carl Zeiss). Percentage of perfused BV was calculated as the lectin+/CD31+ perfused BV area divided by total CD31+BV areas in the putative injury area (1.44 mm^2^) of dura mater.

### Evans Blue leakage assay

Mice were anaesthetized immediately prior to retro-orbital injection of Evans Blue solution (Merck, 1% v/w, 200 µl). Animals were killed 1 h after injection as described above. In order to distinguish vascular leakage in the dura mater from the calvarial bone marrow, dura mater tissues were separated from the calvarial bone after overnight decalcification with 10% ethylenediaminetetraacetic acid prior to immunostaining.

### Treatment of blocking antibody

For VEGFR2 and PDGFRβ blockade, we used VEGFR2- and PDGFRβ-neutralizing antibodies DC101 and APB5^28^, respectively. A hybridoma cell line that produces DC101 was purchased from American Type Culture Collection (ATCC). A hybridoma cell line that produces APB5 was generously provided by Akiyoshi Uemura (Nagoya City University, Japan). Hybridoma cells were grown in serum‐free medium. Recombinant proteins in the supernatants were purified by column chromatography with Protein A agarose gel (Oncogene). After purification, the recombinant proteins were quantified using the Bradford assay and confirmed by Coomassie blue staining after sodium dodecyl sulfate–polyacrylamide gel electrophoresis. DC101 (40 mg/kg) or APB5 (25 mg/kg) and an equal amount of the control antibody (IgG-Fc) was i.p. injected into the indicated mice every other day until mice were sacrificed for analyses.

### Human dura mater and brain samples

Specimens of dura mater and brain cortex obtained from four deceased individuals who suffered fatal HI from vertical falls were autopsied with court-issued warrants at the request of the public prosecutor. The Institutional Review Board of Chonnam National University Medical School and Hospital (Gwangju, Republic of Korea) approved all autopsies as research activity involving non-living, post-mortem subjects in accordance with ethical regulations and waived the requirement to obtain informed consent owing to the inaccessibility to and unavailability of personal identification information and testing for heritable traits of the deceased (no. 2019-001). Accordingly, all specimens and data, with the exception of age, gender, and medical histories, were anonymized prior to all analyses. Dura mater samples were whole-mount immunostained, whereas brain cortex samples were sectioned at 100 µm with a vibrating microtome (Leica Biosystems) prior to immunostaining.

### qRT-PCR analyses of whole dura mater tissue

Dura mater was isolated as mentioned above without any transcardial perfusion. Dura mater was removed from the attaching calvarial bone by submerging the tissue in ice-cold PBS and peeling the organ with fine forceps. Tissue was mechanically dissociated using a Precellys 24 tissue homogenizer (Bertin Technologies) until cells were completely lysed. RNA was extracted using RNeasy Micro kit (Qiagen) or Trizol RNA extraction kit (Invitrogen). A total of 1 µg of extracted RNA was transcribed into cDNA using GoScript Reverse Transcription Kit (Promega). Quantitative real-time PCR was performed using FastStart SYBR Green Master mix (Roche) and S1000 Thermocycler (Bio-Rad) with the indicated primers. The primers were designed using Primer-BLAST or adopted from previously published studies: *Vegfa* (5′*-*TGCCAAGTGGTCCCAGGCTGC-3′; 5′-CCTGCACAGCGCATCAGCGG-3′*); Vegfc* (5′-GAGGTCAAGGCTTTTGAAGGC-3′; 5′-CTGTCCTGGTATTGAGGGTGG-3′); *Pgf* (5′-TCTGCTGGGAACAACTCAACA-3′; 5′-GTGAGACACCTCATCAGGGTAT-3′). *Gapdh* (5′-AGGTCGGTGTGAACGGATTTG-3′; 5′-TGTAGACCATGTAGTTGAGGTCA-3′) was used as a reference gene and the results were presented as relative expressions to control. Primer reaction specificity was confirmed by melting curve analysis. Relative gene expression was analyzed by ΔΔCt method using the CFX Manager software (Bio-Rad).

### ELISA for quantification of VEGF-A

Dura mater was separated from the calvarial bone as described above. Brain cortex was separated from the rest of the brain using a surgical scalpel. Tissues were mechanically dissociated as described above and protein was extracted in Cell Extraction Buffer (ThermoFischer Scientific). Sample protein concentrations were measured and normalized with a Pierce BCA Protein Assay Kit (ThermoFisher Scientific) and the concentration of VEGF-A in tissue extracts was measured using a Mouse VEGF-A Quantikine ELISA Kit (MV00, R&D).

### Depletion of CX3CR1^+^ monocytes/macrophages

To deplete CX3CR1^+^ monocytes and macrophages in CX3CR1-DTR mice receiving PTI, 1 μg of diphtheria toxin (Merck) diluted in PBS was injected every other day, beginning from 1 day before PTI until mice were sacrificed at 7 days after PTI for analysis. Control mice received injections of PBS.

### Spheroid-based sprouting assay

EC spheroids were generated by incubating ∼1000 FACS-sorted primary dura mater and brain ECs in endothelial growth media (EGM2, Lonza) containing 0.25% methylcellulose as hanging-drops overnight^35^. The spheroids were then collected and embedded in 2 mg/ml collagen type I (Corning) with mouse VEGF-A-supplied (50 ng/ml) EGM for 24 h. At the end of the incubation, spheroid was stained with cell tracker (Molecular Probes, 1.5 μM, 37 °C, 30 min). The spheroids were then fixed in 4% PFA for 15 min at RT and imaged using a Zeiss LSM 800 confocal microscope (Carl Zeiss).

### siRNA-mediated knockdown

Human umbilical vein ECs (HUVECs) were maintained in EGM2 media (Lonza). For small RNA interference (siRNA), oligonucleotides against Aqp1 (358-2, Bioneer), against Fabp4 (2167-1, Bioneer), against Esm1 (11082-2, Bioneer), or scrambled siRNA for negative control were used. HUVECs with a 40–60% confluence were transfected with siRNAs using Lipofectamine RNAiMAX (Invitrogen) in OptiMEM media. Culture media was changed 4–6 hr after transfection.

### Magnetic-associated and fluorescence-activated cell sorting

To sort dura mater or brain ECs, CD45^+^ hematopoietic or CD45^−^ non-hematopoietic cells by FACS, tissues were isolated as described above and dissociated in collagenase type I (1 mg/ml, Worthington), dispase (1 mg/ml, Gibco), and DNase I (0.1 mg/ml, Merck) dissolved in DMEM/F12 supplemented with 5% fetal bovine serum (FBS) at 37 °C for 30 min. Cell suspensions went on RBC lysis by suspension in ACK lysis buffer (Gibco) for 5 min on ice and blocked with mouse anti-CD16/CD32 (553141, BD Bioscience) before being incubated for 20 min with indicated antibodies in FACS buffer (2% FBS in PBS). After several washes, brain ECs were enriched using automatic magnetic-associated cell sorting (MACS, Miltenyi-Biotec) prior to be being stained with fluorochrome-conjugated primary antibodies. Cell sorting was performed with FACS Aria II (Beckton Dickinson). Dead cells were excluded using DAPI (Sigma-Aldrich) staining and cell doublets were systematically excluded. The following antibodies were used for MACS: biotin anti-mouse CD31 (rat monoclonal, 130-119-562, Miltenyi-Biotec); anti-biotin microbeads (130-090-485). The following antibodies were used for FACS: PE anti-mouse CD31 (rat monoclonal, 102508, Biolegend); APC anti-mouse CD45 (rat monoclonal, 103112, Biolegend); FITC anti-mouse TER-119 (rat monoclonal, 116206, Biolegend).

### Bulk RNA-sequencing

Bulk RNA-sequencing of sorted ECs was performed by obtaining the alignment file. In brief, reads were mapped using TopHat software tool. The alignment file was used to assemble transcripts, estimate their abundances, and detect differential expression of genes or isoforms using cufflinks. The Ingenuity Pathway Analysis tool (QIAGEN) was used to further evaluate the data in the context of biological categories and canonical signaling. The significance of biological categories and canonical signaling was tested by the Benjamini–Hochberg procedure, which adjusts the *P* value to correct for multiple comparisons, and their activation or inhibition was determined with reference to activation *z* scores.

### Parabiosis model

Pairs of 8–10-week-old wild-type and actin-GFP^+^ mice were subjected to parabiotic surgery. Mice were anesthetized by intramuscular injection of the combination of anesthetics, and then surgically joined from shoulder to femur. At the indicated weeks after parabiotic surgery, GFP^+^ cells in the injured tissues of wild-type mice were examined.

### Single-cell-RNA sequencing using droplet-based method

Single-cell suspensions were prepared as described above, diluted to 1000 cells/μl in PBS, and processed with the 10× Chromium platform using 10× Chromium single-cell 3′ reagents, following manufacturer’s protocols. In brief, required number of cells were loaded to 10× chips with RT reagent mix and RT primers. After partitioning cells into Gel Beads in Emulsion, transcripts from single cells were barcoded and reverse-transcribed to single-cell cDNA libraries. Then, cDNA libraries were enzymatically fragmented, end-repaired and A-tailed. After double-sized size selection using SPRI beads (Beckman Coulter), adapters were ligated and sample-index PCR was performed. Products went through another round of double-sized size selection using SPRI beads. For quality check of final libraries, they were diluted to 10-fold and run on Agilent Bioanalyzer High Sensitivity Chip. Single-cell libraries that passed quality control were then sequenced on Illumina HiSeq-X platform.

### Construction of raw expression matrix

Raw base call (BCL) files from Illumina sequencers were first demultiplexed into FASTQ files using mkfastq function in Cell Ranger 3.1.0 toolkit from 10X Genomics. Then, reads were mapped to mm10 reference genome (v3.0.0, 10X Genomics). Next, using R package ‘Seurat’ (v3.1.0)^52^, raw expression matrices were built from aligned reads by Read10X function in Seurat. To remove potential doublets formed by cells with distinct transcriptome, Scrublet (Single-Cell Remover of Doublets)^53^ was used. Scrublet generates simulated doublets from randomly selected cells in the data set and constructs nearest neighbor classifier to identify potential doublets. In addition, cells that have high percentage of mitochondrial unique molecular identifier (UMI) counts (>7%), and cells that have too low or too high number of detected genes (>1000 and >6000) were discarded from the data sets. Only cells that passed such stringent quality control processes were used for further analysis.

### Integrating data sets and clustering analysis

From the four data sets, 2500 cells that all passed quality controls, were random sampled, yielding 10,000 cells in total. To observe whether batch effects exist, random sampled cells were first merged without any imputation-based methods. After initial clustering, clusters were not separated by batches, suggesting minimal effect arising from experimental batches. Then, the expression matrix of merged data set was first normalized by the NormalizeData function in Seurat, which normalizes the read counts by dividing UMI counts for each gene in a given cell by the sum of all UMI counts in that cell, multiplies 10,000 and transforms into log scales. Genes that are highly variable in the data set were identified by FindVariableFeatures function in Seurat with options (selection.method = “vst”, nfeatures = 2500). Data were scaled and variables such as mitochondrial percentages and total number of UMI counts were regressed by ScaleData function in Seurat. Dimensionality of the data was reduced by performing principal component analysis with the highly variable genes identified on the scaled data. Top 30 principal components were used for uniform manifold approximation and projection to visualize data into two-dimensional space. For the identification of clusters, FindNeighbors and FindClusters functions in Seurat were used with 30 principal components and with resolution parameter of 0.6, respectively.

### Identifying differentially expressed genes

Cluster-specific DEG were identified using the FindMarkers function in Seurat. The test MAST (Model-based Analysis of Single-cell Transcriptomics)^54^ was used for differential expression testing, which utilizes the hurdle model fit for single-cell data. Top 100 DEGs for each cluster in the integrated data set were used as input in Immgen (http://www.immgen.org) data browser to characterize and annotate cell populations. Finally, to visualize DEGs identified between clusters, DoHeatmap function for heatmaps and VlnPlot function for violin plots in Seurat were used with default parameters.

### Statistical analysis

No statistical methods were used to predetermine sample size. The experiments were randomized and investigators were blinded to allocation during experiments and outcome analyses. All values are presented as mean ± standard deviation (SD). Statistical significance was determined by the two-sided Mann–Whitney *U* test between two groups or the two-way analysis of variance by Kruskal–Wallis multiple comparisons test for multiple-group comparison. Statistical analyses were performed using GraphPad Prism 8.0 (GraphPad Software). Statistical significance was set at *P* < 0.05.

### Reporting summary

Further information on research design is available in the Nature Research Reporting Summary linked to this article.

## Supplementary information

Supplementary Information

Reporting Summary

## Data Availability

RNA-sequencing data are available in the National Center for Biotechnology Information’s Gene Expression Omnibus under accession numbers GSE145973 (scRNAseq) [https://www.ncbi.nlm.nih.gov/geo/query/acc.cgi?acc=GSE145973] and GSE138561 (bulk RNAseq) [https://www.ncbi.nlm.nih.gov/geo/query/acc.cgi?acc=GSE138561]. All other data that support the findings of this study are available from the corresponding author upon reasonable request. Source data are provided with this paper.

## Source data

Source Data
