# Supplementary material for: VEGFR2 signaling drives meningeal vascular regeneration upon head injury
Source: Nat Commun. 2020 Jul 31;11:3866. doi: 10.1038/s41467-020-17545-2 (PMC7395111; doi:10.1038/s41467-020-17545-2)
Supplement: Supplementary file 1 — Supplementary Information [file 41467_2020_17545_MOESM1_ESM.pdf]

Supplementary Information

VEGFR2 signaling drives meningeal vascular regeneration upon head injury

Bong Ihn Koh, Hyuek Jong Lee, Pil Ae Kwak, Myung Jin Yang, Ju-Hee Kim, Hyung-Seok Kim, Gou Young Koh, Injune Kim

Includes:

1. Supplementary Figures 1-15

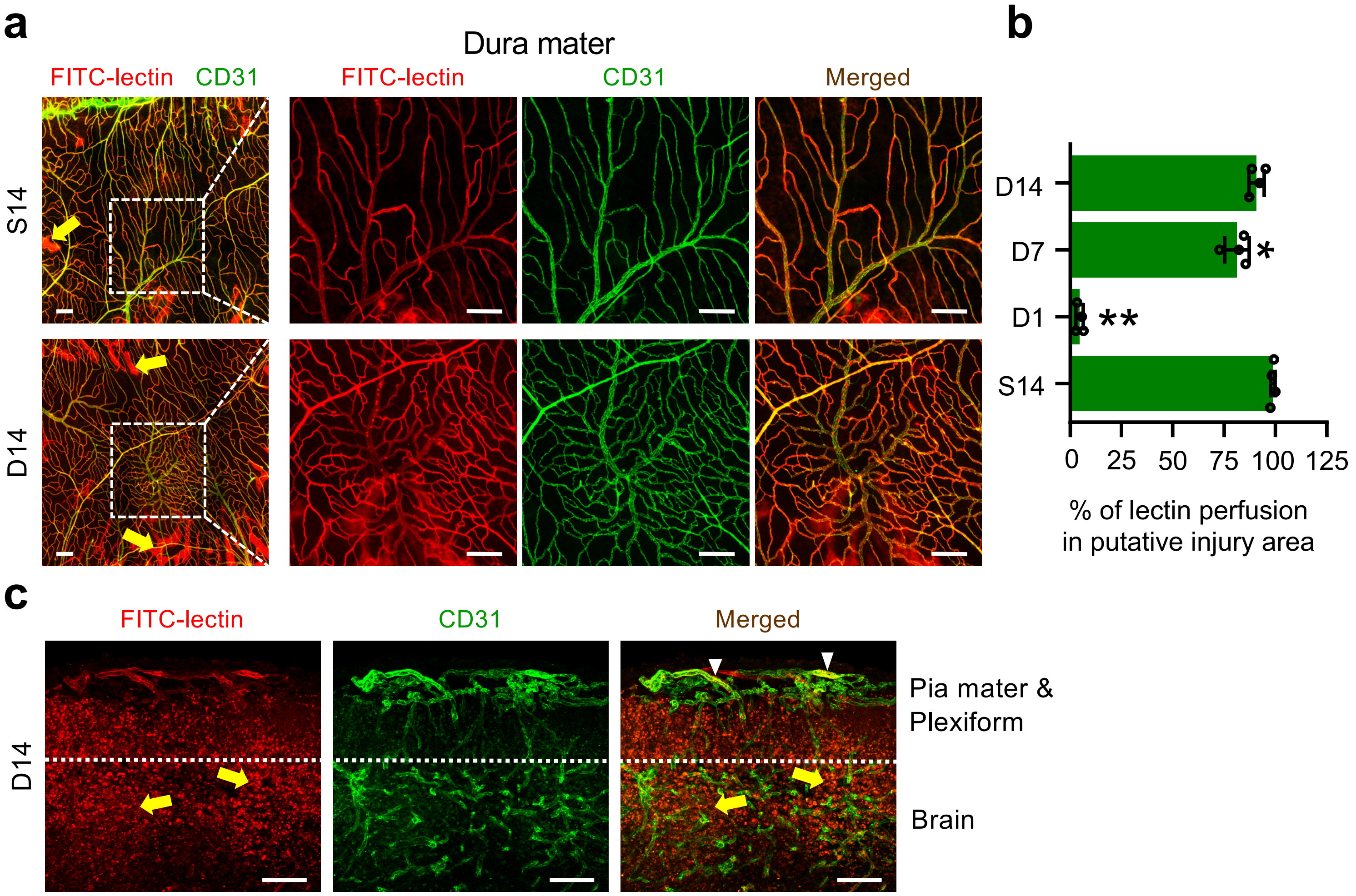

Supplementary Figure 1. Rapid recovery of vascular perfusion in the injured dura mater but not injured brain after PTI. a, **b**, Representative images and comparisons of blood perfusion into CD31+ BVs in putative injury area (white dotted-lined box) at indicated days in the dura mater of adult mice after PTI. Each box is magnified and presented as right three panels. Yellow arrows indicate leakage of FITC-lectin into bone marrow of skull. Scale bars, 200 μm. Each dot indicates a mean value obtained from one mouse and *n* = 4 mice/group from two independent experiments. Mean fluorescence intensity (MFI) of FITC-lectin per MFI of CD31 in the putative injury area at S14 is regarded as 100%. Horizontal bars indicate mean ± SD. **P* <0.05 and ***P* <0.01 versus S7 by Kruskal-Wallis test. **c**, Representative images of poor blood perfusion into CD31+ BVs in the injury core area at D14 in the brain of adult mice after PTI. Yellow arrows indicate leakage of FITC-lectin into injured brain parenchyma. White arrowheads indicate FITC-lectin-perfused vessels in the pia mater. Similar findings were observed in *n* = 3 mice from two independent experiments. Scale bars, 100 μm.

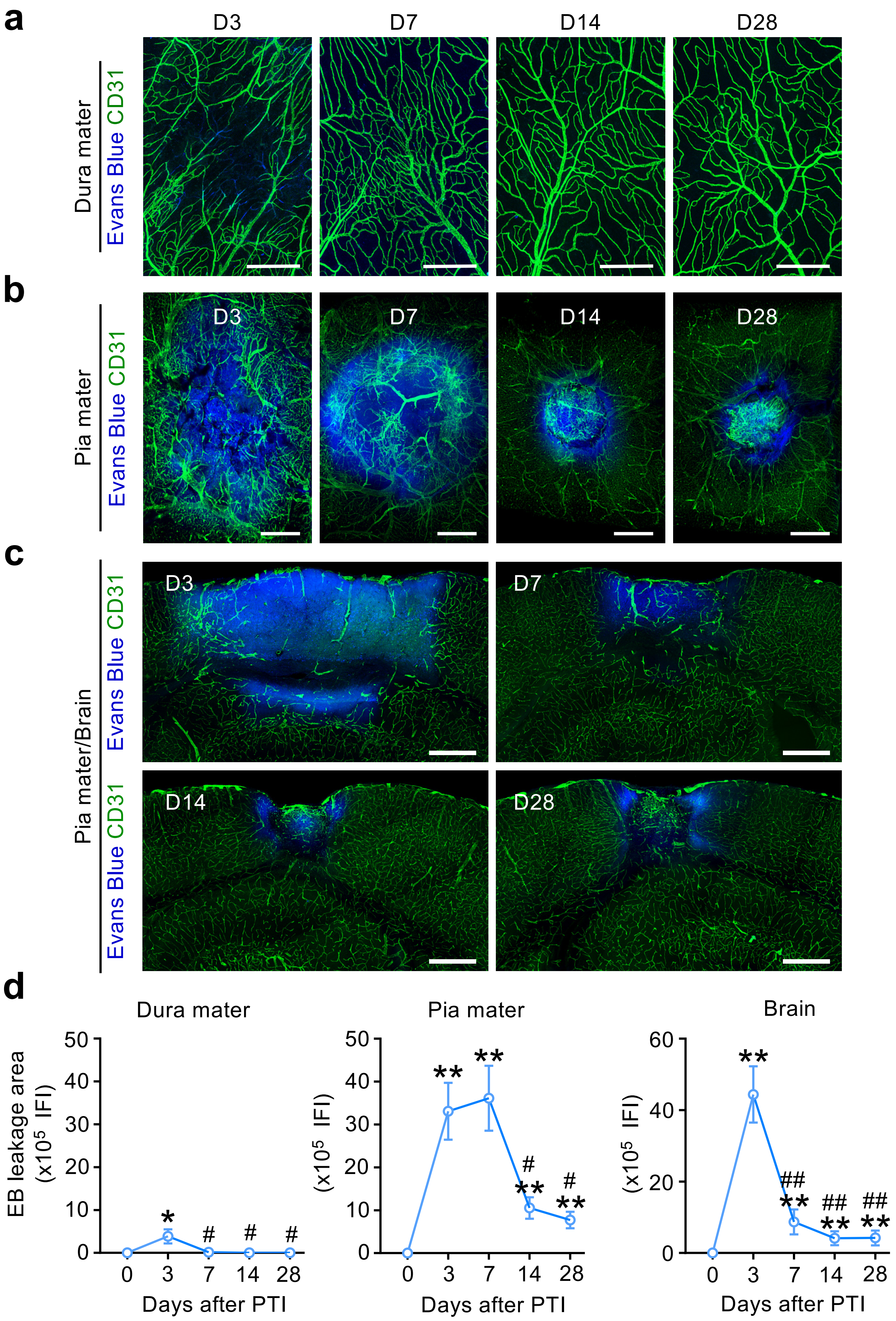

Supplementary Figure 2. Relatively low and transient vascular leakage in the injury area of dura mater compared to pia mater and brain after PTI. a-d, Representative images and comparisons of Evans Blue (EB) leakage in the putative injury area of dura mater, pia mater and brain of adult mice at indicated days after PTI. Scale bars, 500 µm. Each dot indicates a value obtained from a mouse and *n* = 4 mice/group from two independent experiments. Dots and error bars indicate mean ± SD. $*P<0.05$ and $**P<0.01$ versus P0, $^{\#}P<0.05$ and $^{\#\#}P<0.01$ versus D3 by Kruskal-Wallis test. IFI, integrated fluorescence intensity.

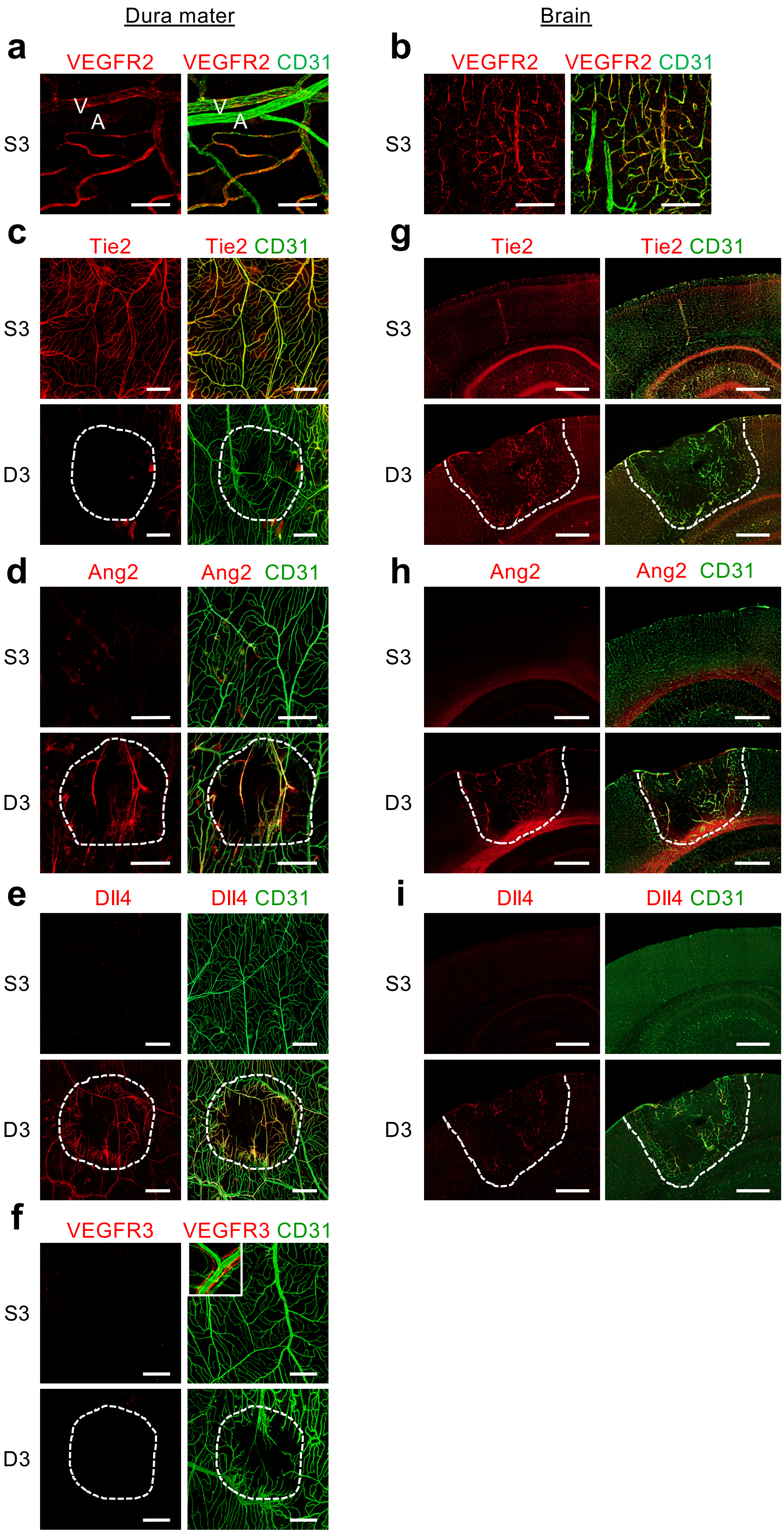
Dura mater
Brain
a
VEGFR2
VEGFR2 CD31
V
A
S3
b
VEGFR2
VEGFR2 CD31
S3
c
Tie2
Tie2 CD31
S3
D3
g
Tie2
Tie2 CD31
S3
D3
d
Ang2
Ang2 CD31
S3
D3
h
Ang2
Ang2 CD31
S3
D3
e
Dll4
Dll4 CD31
S3
D3
i
Dll4
Dll4 CD31
S3
D3
f
VEGFR3
VEGFR3 CD31
S3
D3

Supplementary Figure 3. Molecular changes in dura mater and brain BVs in response to PTI. **a**, **b**, Representative images of VEGFR2 localization in CD31+ BVs in the dura mater and brain of adult mice. V, venule; A, arteriole. Similar findings were observed in n = 3 mice from two independent experiments. Scale bars, 100 µm. **c**-**f**, Representative images of changes of Tie2, Ang2, Dll4 and VEGFR3 in CD31+ BVs in the putative injury area (white dotted-lined circle) of dura mater of adult mice at S3 and D3. VEGFR3+ meningeal lymphatic vessel is shown as a positive control in white-lined box. Similar findings were observed in n = 3 mice from two independent experiments. Scale bars, 500 µm. **g**-**i**, Representative images of changes of Tie2, Ang2 and Dll4 in CD31+ BVs in the putative injury area (white dotted line) of brain of adult mice at S3 and D3. Similar findings were observed in n = 3 mice from two independent experiments. Scale bars, 500 µm.

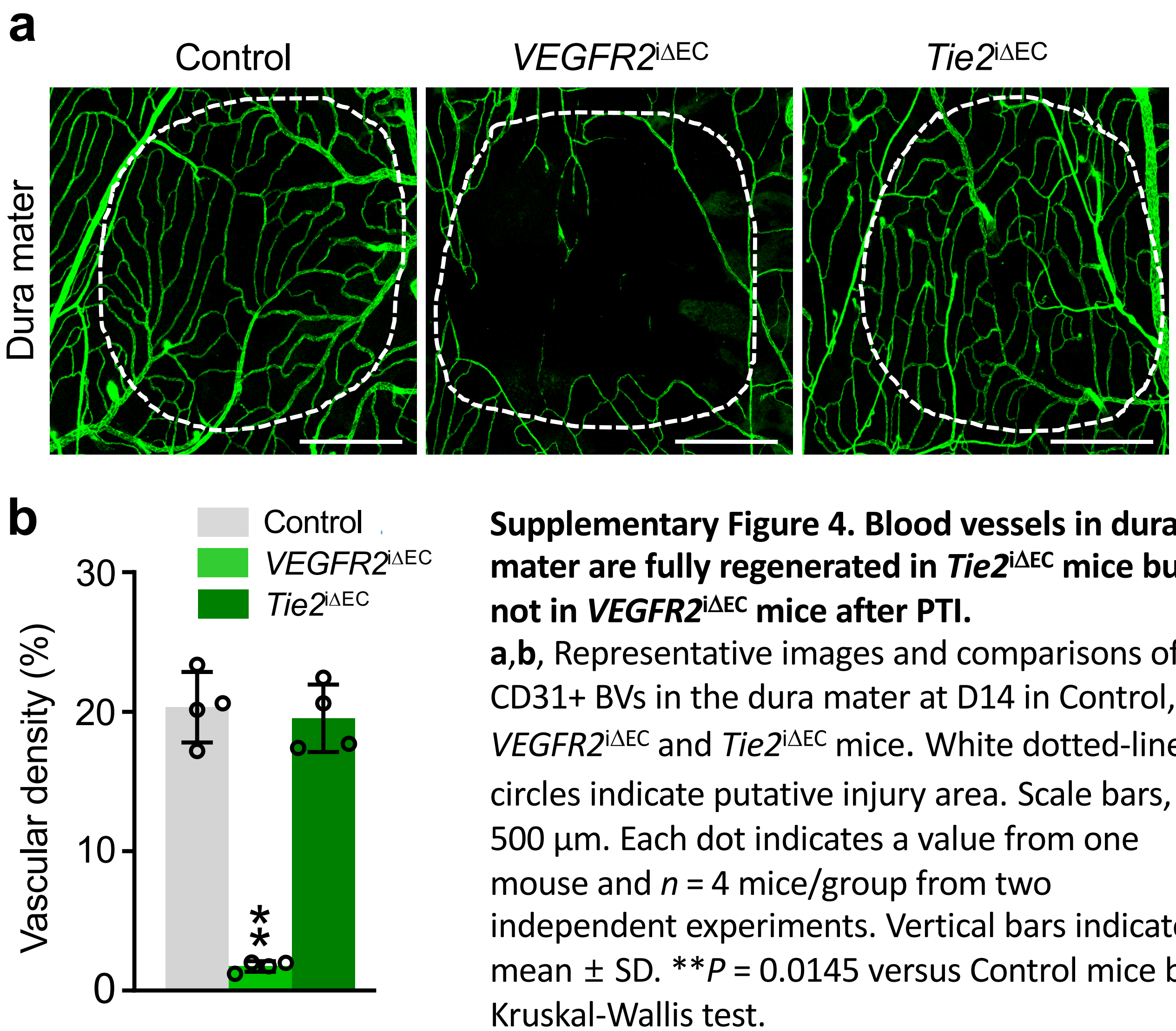

Supplementary Figure 4. Blood vessels in dura mater are fully regenerated in *Tie2*$^{i\Delta EC}$ mice but not in *VEGFR2*$^{i\Delta EC}$ mice after PTI.
a,**b**, Representative images and comparisons of CD31+ BVs in the dura mater at D14 in Control, *VEGFR2*$^{i\Delta EC}$ and *Tie2*$^{i\Delta EC}$ mice. White dotted-lined circles indicate putative injury area. Scale bars, 500 µm. Each dot indicates a value from one mouse and n = 4 mice/group from two independent experiments. Vertical bars indicate mean ± SD. ***P* = 0.0145 versus Control mice by Kruskal-Wallis test.

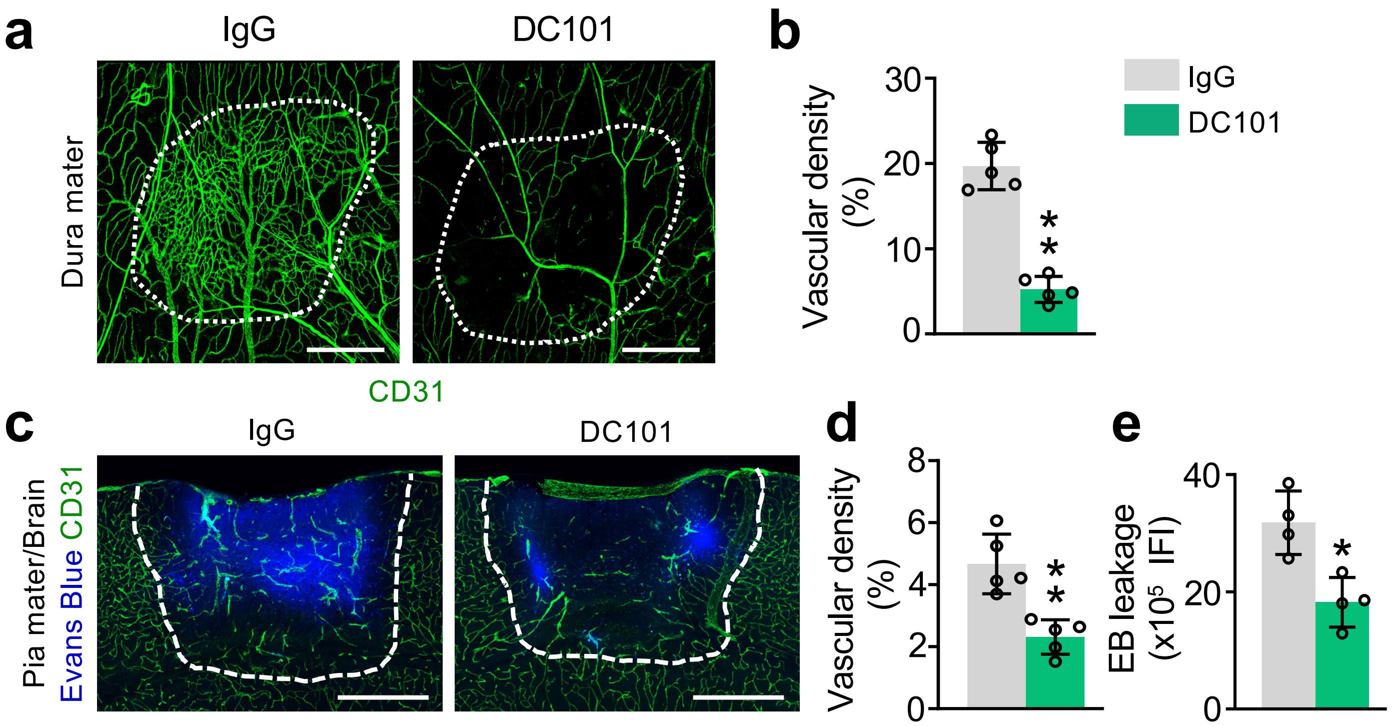

Supplementary Figure 5. VEGFR2 signaling is critical for vascular regeneration in the dura mater and brain after PTI. a-e, Representative images and comparisons of CD31+ BVs in the dura mater and brain, and Evans Blue (EB) leakage in brain at D7 in adult mice that were treated with IgG-Fc or DC101 (daily i.p. injection of 40 mg/kg of body weight for 6 days). White dotted-lined circle or line indicates putative injury area. Scale bars, 200 μm. Each dot indicates a mean value obtained from one mouse and *n* = 5 (**b**, **d**), *n* = 4 (**e**) mice/group from two independent experiments. Vertical bars indicate mean ± SD. *P = 0.0286, **P = 0.0079 versus IgG-Fc by two-tailed Mann-Whitney *U* test. IFI, integrated fluorescence intensity.

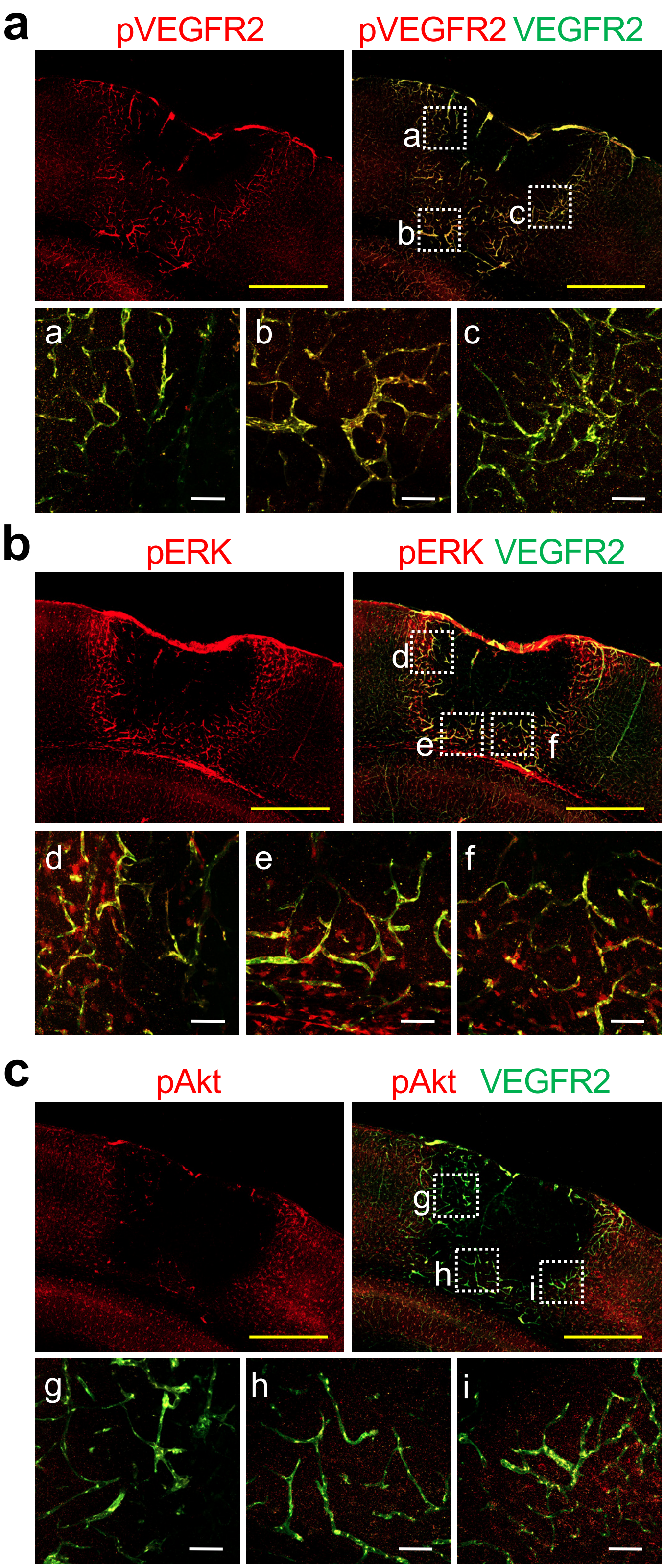

Supplementary Figure 6. Dynamic changes in VEGFR2 and its downstream signaling pathways during vascular remodeling in the brain after PTI. a-c, Representative images of phosphorylated VEGFR2, ERK and Akt in regenerating BVs in the brain of adult mice at D3. Each box region is magnified and presented in the lower panels. Note full coupling of VEGFR2 with ERK phosphorylation but partial coupling with Akt phosphorylation in the EC sprouts of vascular front. Scale bars, 50 µm (white) and 500 µm (yellow). Similar findings were observed in n = 4 mice from two independent experiments.

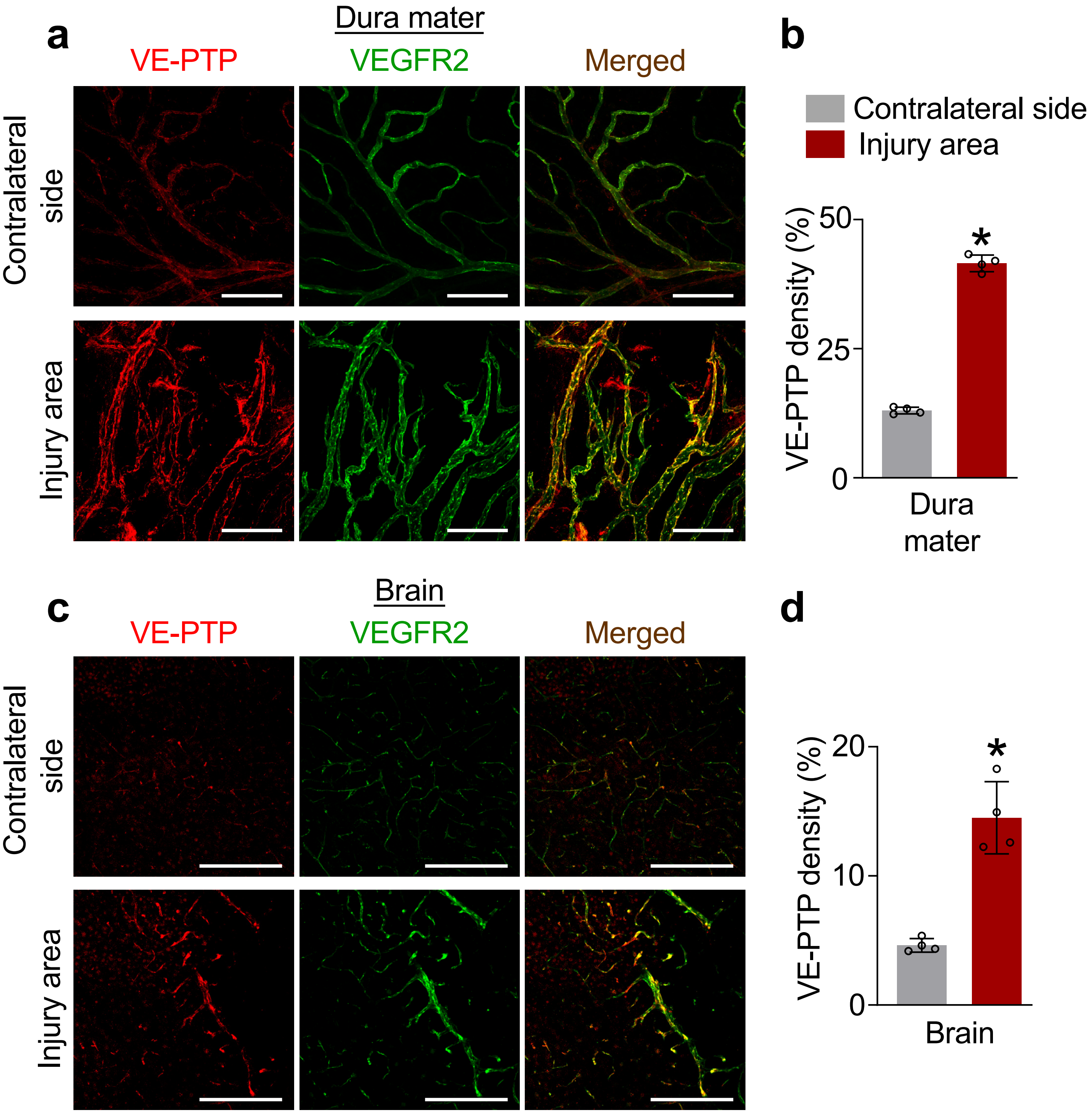

Supplementary Figure 7. Increase of VE-PTP in VEGFR2+ BVs in dura mater and brain in response to PTI. a-d, Representative images and comparisons of VE-PTP in VEGFR2+ BVs in the injury area and contralateral (CL) side of dura mater (**a**, **b**) and brain (**c**, **d**) of adult mice at D3 after PTI. Scale bars, 200 µm. Each dot indicates a value from one mouse and $n = 4$ mice/group from two independent experiments. Vertical bars indicate mean ± SD. *$P = 0.0286$ versus CL by two-tailed Mann-Whitney *U* test.

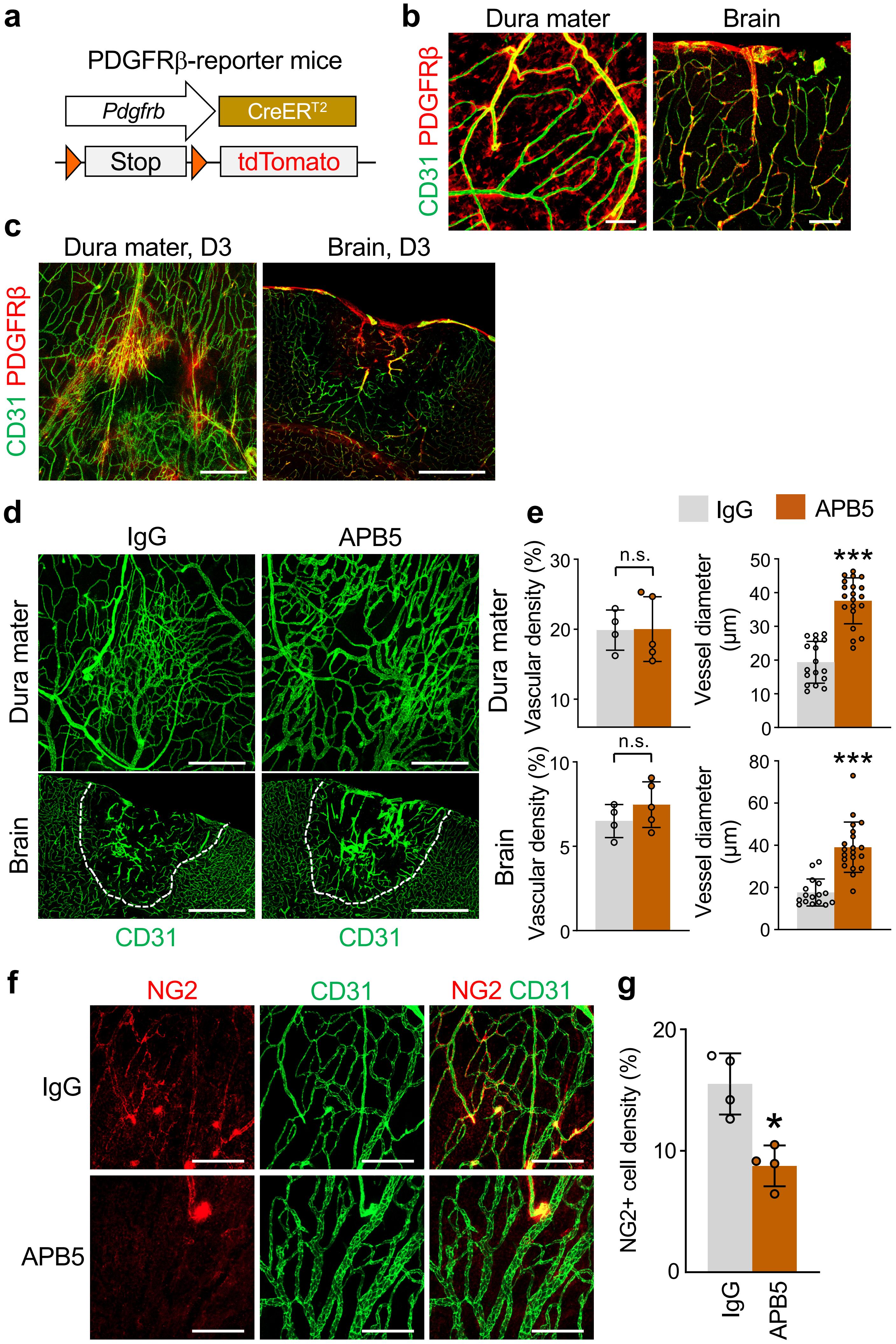
a
PDGFRβ-reporter mice
Pdgfrb
CreERT2
Stop
tdTomato
b
Dura mater
Brain
CD31 PDGFRβ
c
Dura mater, D3
Brain, D3
CD31 PDGFRβ
d
IgG
APB5
Dura mater
Brain
CD31
CD31
e
IgG
APB5
Dura mater
Vascular density (%)
n.s.
Vessel diameter (μm)
***
Brain
Vascular density (%)
n.s.
Vessel diameter (μm)
***
f
NG2
CD31
NG2 CD31
IgG
APB5
g
NG2+ cell density (%)
*
IgG
APB5

Supplementary Figure 8. PDGFRβ+ pericytes play stabilizing role in vascular regeneration in dura mater and brain after PTI. a, Schematic diagram for generation of PDGFRβ-reporter mice. **b**, **c**, Representative images of distribution of PDGFRβ+ pericytes and fibroblasts in dura mater and brain cortex at normal state using PDGFRβ-Cre^{ERT2}-tdTomato reporter mice (**b**) at D3 by IHC (**c**). Similar findings were observed in 3 mice from two independent experiments. Scale bars, 100 µm (**b**) and 500 µm (**c**). **d**, **e**, Representative images and comparisons of CD31+ BVs in the dura mater and brain at D7 in adult mice that were treated with IgG or APB5 (daily i.p. injection of 25 mg/kg of body weight for 6 days). Note enlarged regenerated vessels in the injury areas with APB5 treatment. Scale bars, 500 µm. Each dot indicates a value from one mouse for vascular density, while 3-5 (IgG) or 3 (APB5) values were obtained from one mouse for vessel diameter. n = 4 (IgG), n = 5 (APB5) mice/group from two independent experiments. Vertical bars indicate mean ± SD. ***P <0.001 versus IgG-Fc by two-tailed Mann-Whitney U test. **f**, **g**, Representative images and comparison of distribution of NG2+ pericytes along regenerated blood vessels in the injury core area of dura mater at D7 after PTI. Note reduced number and detachment of NG2+ pericytes are accompanied with enlarged regenerated blood vessels in dura mater after PTI with APB5 treatment. Scale bars, 200 µm. n = 4 mice/group from two independent experiments. Vertical bars indicate mean ± SD. *P = 0.0286 versus IgG-Fc by two-tailed Mann-Whitney U test.

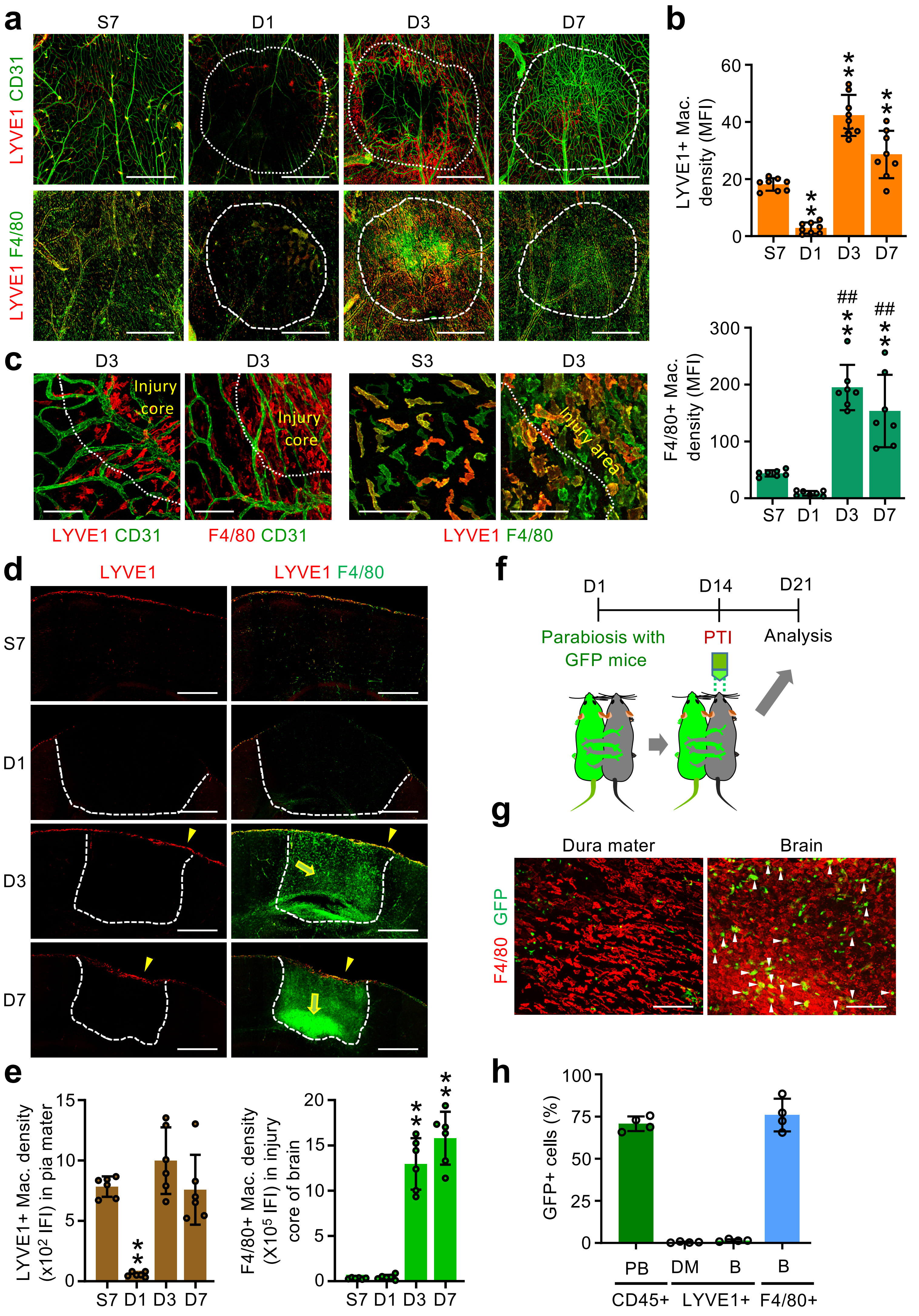
a
S7
D1
D3
D7
LYVE1 CD31
LYVE1 F4/80
b
LYVE1+ Mac. density (MFI)
60
40
20
0
S7 D1 D3 D7
F4/80+ Mac. density (MFI)
300
200
100
0
##
c
Injury core
Injury area
S3
LYVE1 CD31
F4/80 CD31
LYVE1 F4/80
d
LYVE1
LYVE1 F4/80
e
LYVE1+ Mac. density (x10² IFI) in pia mater
15
10
5
0
F4/80+ Mac. density (X10⁵ IFI) in injury core of brain
20
f
D1
D14
D21
Parabiosis with GFP mice
PTI
Analysis
g
Dura mater
Brain
F4/80 GFP
h
GFP+ cells (%)
100
75
50
25
PB
DM
B
CD45+
LYVE1+
F4/80+

Supplementary Figure 9. Dynamic changes of distributions of LYVE1+ and F4/80+ macrophages in the injury areas of dura mater and brain after PTI.

a, **b**, Representative images and comparisons of the distributions of LYVE1+ and F4/80+ macrophages in the putative injury area (white dotted-lined circle) of dura mater of adult mice at indicated days after PTI. Scale bars, 1 mm. Each dot indicates a mean value obtained from one mouse and *n* = 8 (LYVE1+), *n* = 7 (F4/80+) mice/group from three independent experiments. Vertical bars indicate mean ± SD. **P* <0.05 and ***P* <0.01 versus S7, $^{\#}P$ <0.05 and $^{\#\#}P$ <0.01 versus D1 by Kruskal-Wallis test. MFI, mean fluorescence intensity. **c**, Representative images showing moderately accumulated LYVE1+ macrophages at the leading edge and highly accumulated F4/80+ macrophages within the core of injury area, and their morphological changes in the dura mater at D3 in adult mice. White dotted lines indicate the putative injury margins. Similar findings were observed in *n* = 4 mice from two independent experiments. Scale bars, 100 µm. **d**, **e**, Representative images and comparisons of the distributions of LYVE1+ and F4/80+ macrophages in the putative injury area (white dotted line) at indicated days in the pia mater and brain after PTI in adult mice. Yellow arrowheads indicate confined distributions of LYVE1+ macrophages to pia mater, while green arrowheads indicate highly accumulated F4/80+ macrophages in the injured brain parenchyma. Scale bars, 500 µm. Each dot indicates a mean value obtained from one mouse and *n* = 6 mice/group from two independent experiments. Vertical bars indicate mean ± SD. ***P* <0.01 versus S7 by Kruskal-Wallis test. IFI, integrated fluorescence intensity. **f**-**h**, Timeline for parabiosis with actin-GFP+ mice at day 1 (D1), PTI at D14 and analyses at D21. Images and comparisons of distributions of GFP+ cells at 21 days after parabiosis and 7 days after PTI in the injury areas of dura mater (DM) and brain (B), and CD45+ cells of peripheral blood (PB). Scale bars, 500 µm. Each dot indicates a mean value obtained from one mouse and *n* = 4 mice/group from two independent experiments. Vertical bars indicate mean ± SD.

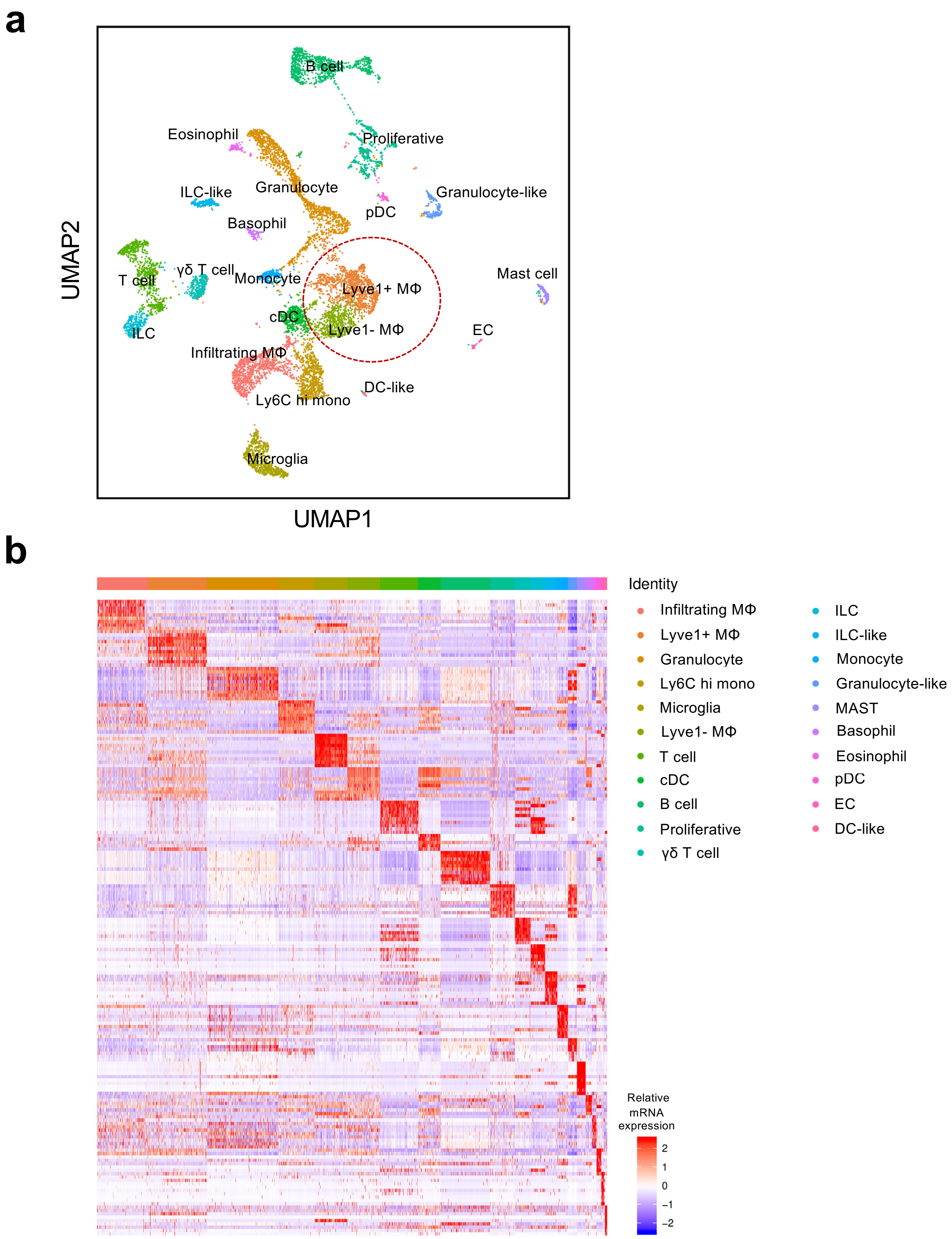

Supplementary Figure 10. Single cell RNA sequencing reveals 21 distinct immune cell subsets in dura mater and brain under sham and PTI conditions. **a**, Uniform manifold approximation and projection (UMAP) visualization of total 10,000 cells from single-cell analysis of FACS-sorted CD45+ hematopoietic cells from dura mater or brain at S3 and D3. 2,500 cells were randomly sampled from each tissue and condition. Note Lyve1+ and Lyve1- macrophage subclusters (red dotted circle). **b**, Heatmap showing relative expression of top 10 marker genes identified for each SCA cluster.

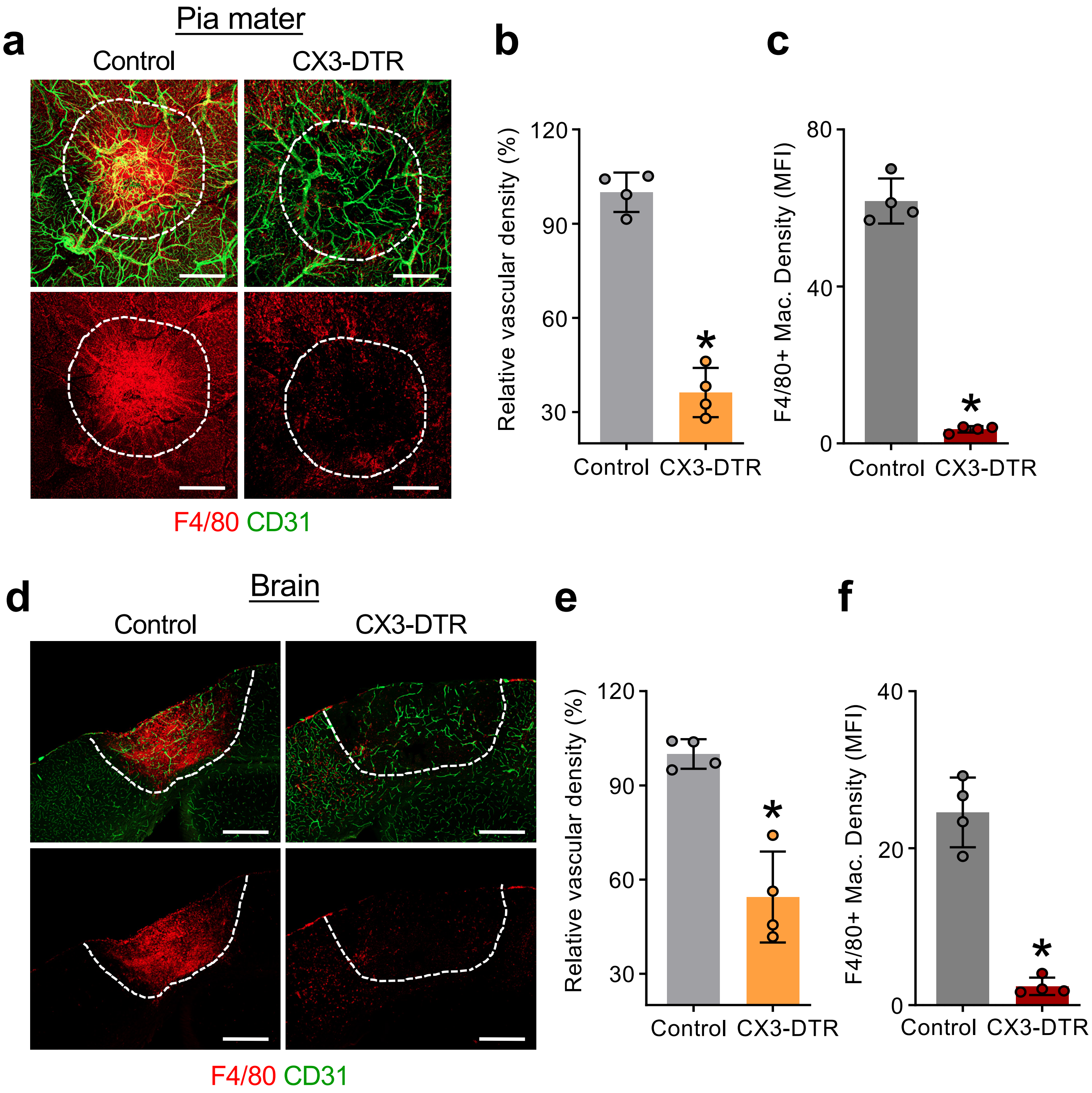

Supplementary Figure 11. Macrophages are required for vascular regeneration in pia mater and brain. a-f, Representative images and comparisons of vascular regeneration and distribution of F4/80+ macrophages in the pia mater and brain between Control and CX3CR1-DTR (CX3-DTR) mice at D7 after PTI. White dotted-lined circles and lines indicate putative injury area. Scale bars, 500 µm. Each dot indicates a mean value obtained from one mouse and *n* = 4 mice/group from two independent experiments. Vertical bars indicate mean ± SD. *P = 0.0286 versus Control by two-tailed Mann-Whitney *U* test. MFI, mean fluorescence intensity.

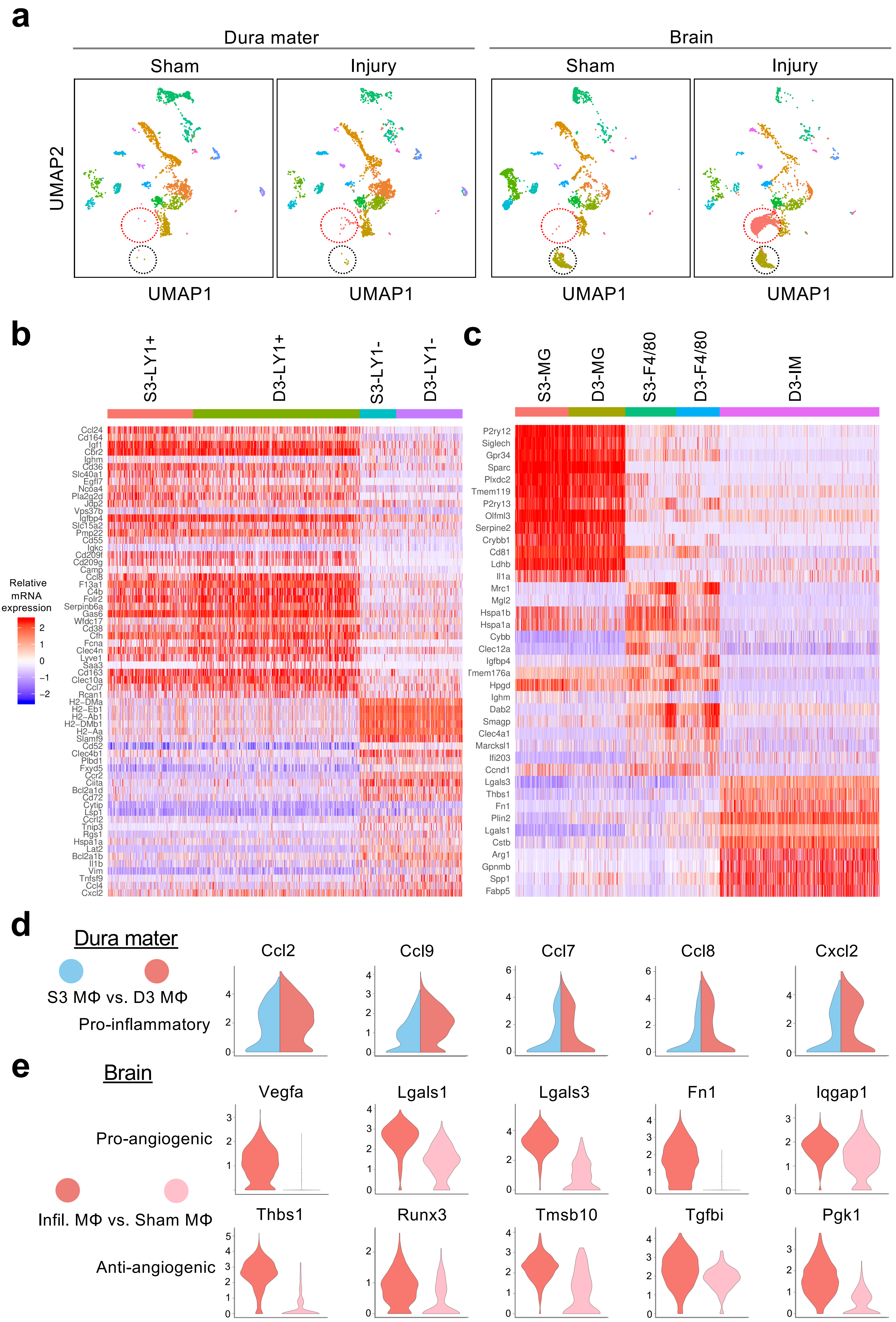

a
Dura mater
Brain
Sham
Injury
Sham
Injury
UMAP2
UMAP1
UMAP1
UMAP1
UMAP1
b
S3-LY1+
D3-LY1+
S3-LY1-
D3-LY1-
Relative mRNA expression
c
S3-MG
D3-MG
S3-F4/80
D3-F4/80
D3-IM
d
Dura mater
S3 MΦ vs. D3 MΦ
Pro-inflammatory
Ccl2
Ccl9
Ccl7
Ccl8
Cxcl2
e
Brain
Pro-angiogenic
Infil. MΦ vs. Sham MΦ
Anti-angiogenic
Vegfa
Lgals1
Lgals3
Fn1
Iqgap1
Thbs1
Runx3
Tmsb10
Tgfbi
Pgk1

Supplementary Figure 12. Single cell RNA sequencing shows minor changes in dura mater but substantial changes in brain macrophages. **a**, UMAP plot showing CD45+ hematopoietic cell clusters in dura mater and brain at D3. Note microglia cluster (black dotted circle) and emergence of infiltrating macrophages after PTI (red dotted circle), both specific to the brain. **b**, Heatmap of the 20 most differentially expressed genes in Lyve1+ (LY1+) or Lyve1- (LY1-) macrophages of the dura mater at D3. Note minimal changes between sham (S3) and PTI (D3) conditions. High enrichment of MHC class II molecules (*H2-Dma*, *H2-Eb1*, *H2-Ab1*, *H2-DMb1* and *H2-Aa*) is observed in the LY1- macrophage subsets. **c**, Heatmap of distinct clustering of brain-resident microglia (MG), F4/80+ macrophages, and brain-infiltrating macrophages (IM) at D3. **d**, Representative violin plots of up-regulated pro-inflammatory genes in dura mater F4/80+ macrophages at D3 compared to S3. Y-axis shows log-normalized expression levels. All differences were significant with $P < 0.001$ by Wilcoxon rank-sum test with Benjamini-Hochberg correction for multiple testing. **e**, Representative violin plots showing high enrichment of pro- and anti-angiogenic genes in brain-infiltrating (infil.) macrophages compared to brain F4/80+ macrophages from sham-operated control mice. Y-axis shows log-normalized expression levels. All differences were significant with $P < 0.001$ by Wilcoxon rank-sum test with Benjamini-Hochberg correction for multiple testing.

a

Function	Activation z-score	Predicted Activation State	p-value	#Molecules
Vasculogenesis	3.998	Increased	1.64E-21	150
Development of vasculature	3.629	Increased	4.66E-22	191
Angiogenesis	3.621	Increased	7.84E-22	174
Cell movement of endothelial cells	3.459	Increased	6.48E-12	72
Endothelial cell development	3.366	Increased	3.45E-08	62
Development of endothelial tissue	3.34	Increased	1.36E-08	64
Migration of endothelial cells	3.221	Increased	1.40E-10	65
Migration of vascular endothelial cells	3.163	Increased	2.46E-10	40
Movement of vascular endothelial cells	3.054	Increased	7.19E-10	42
Formation of blood vessel	2.725	Increased	2.72E-08	34
Growth of blood vessel	2.066	Increased	2.89E-09	24
Neovascularization of organ	1.457		6.70E-08	26
Neovascularization	1.276		2.46E-10	38
Vascularization	1.232		8.87E-10	47
Vascularization of absolute anatomical region	1.102		6.14E-09	31
Tubulation of endothelial cells	0.771		1.40E-08	28
Vascularization of eye	0.701		1.92E-08	27

b

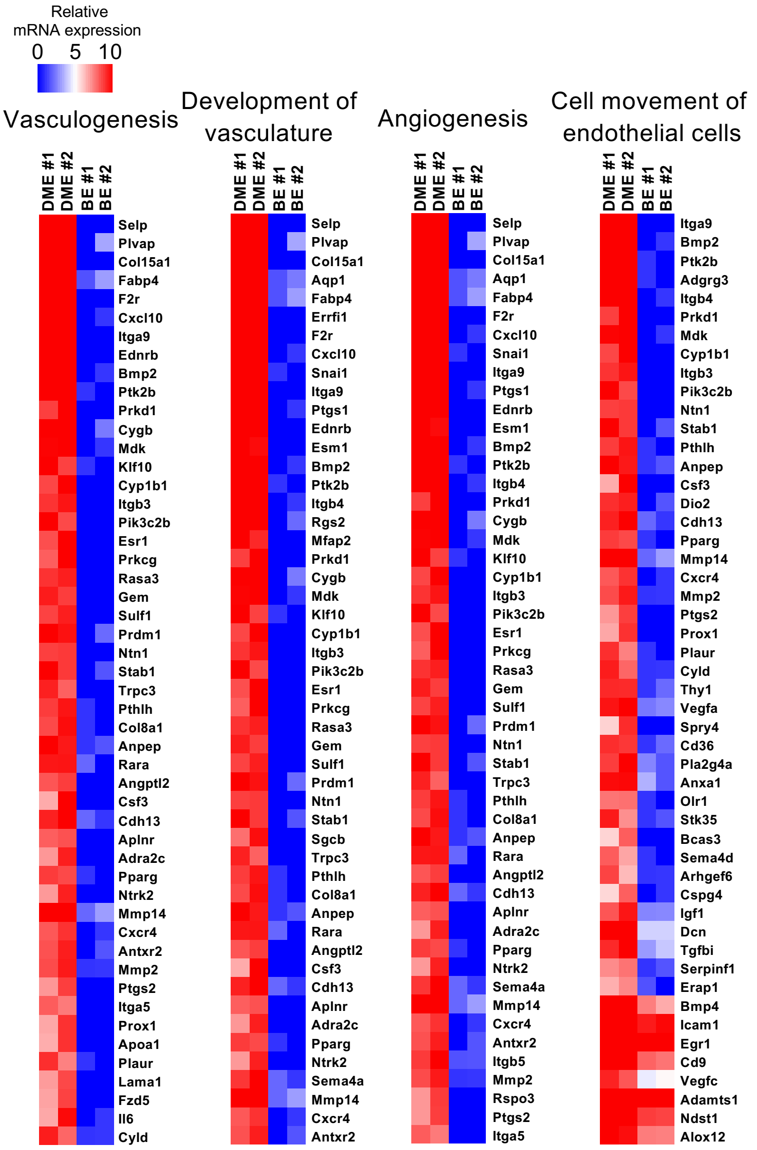

c

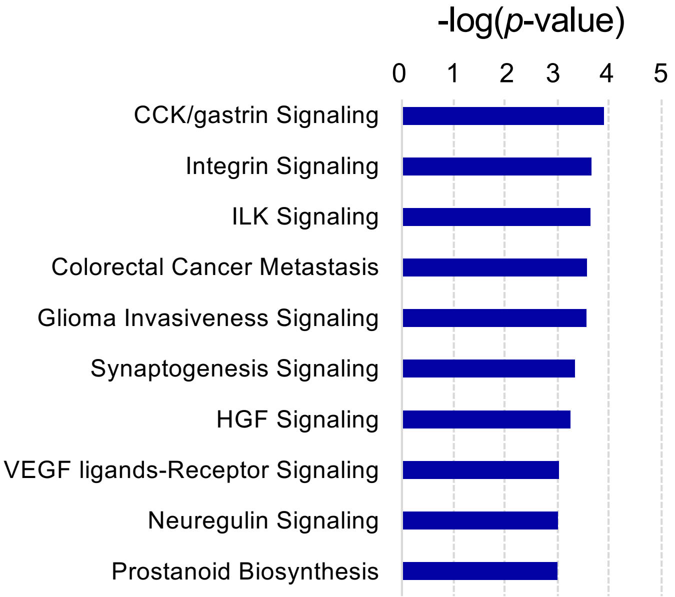

Supplementary Figure 13. High enrichment of angiogenic molecules in dura mater compared to brain ECs.
a, Table of top Cardiovascular System Development and Function pathways from IPA analyses of differentially regulated genes between FACS-sorted primary dura mater ECs and brain ECs. Predicted activation state was determined with reference to activation z-scores with a ±2.0 threshold and significance was tested by right-tailed Fisher's Exact Test with Benjamini-Hochberg correction for multiple testing.
b, Heatmaps of top 50 enriched genes in top Cardiovascular System Development and Function pathways of dura mater ECs (DME) compared to brain ECs (BE). **c**, IPA of canonical pathways from 1,343 differentially expressed genes in DME compared to BE. Ranked according to –log(p-value) within all Ingenuity canonical pathways. *P* <0.001 for all pathways by right-tailed Fisher's Exact Test with Benjamini-Hochberg correction for multiple testing.

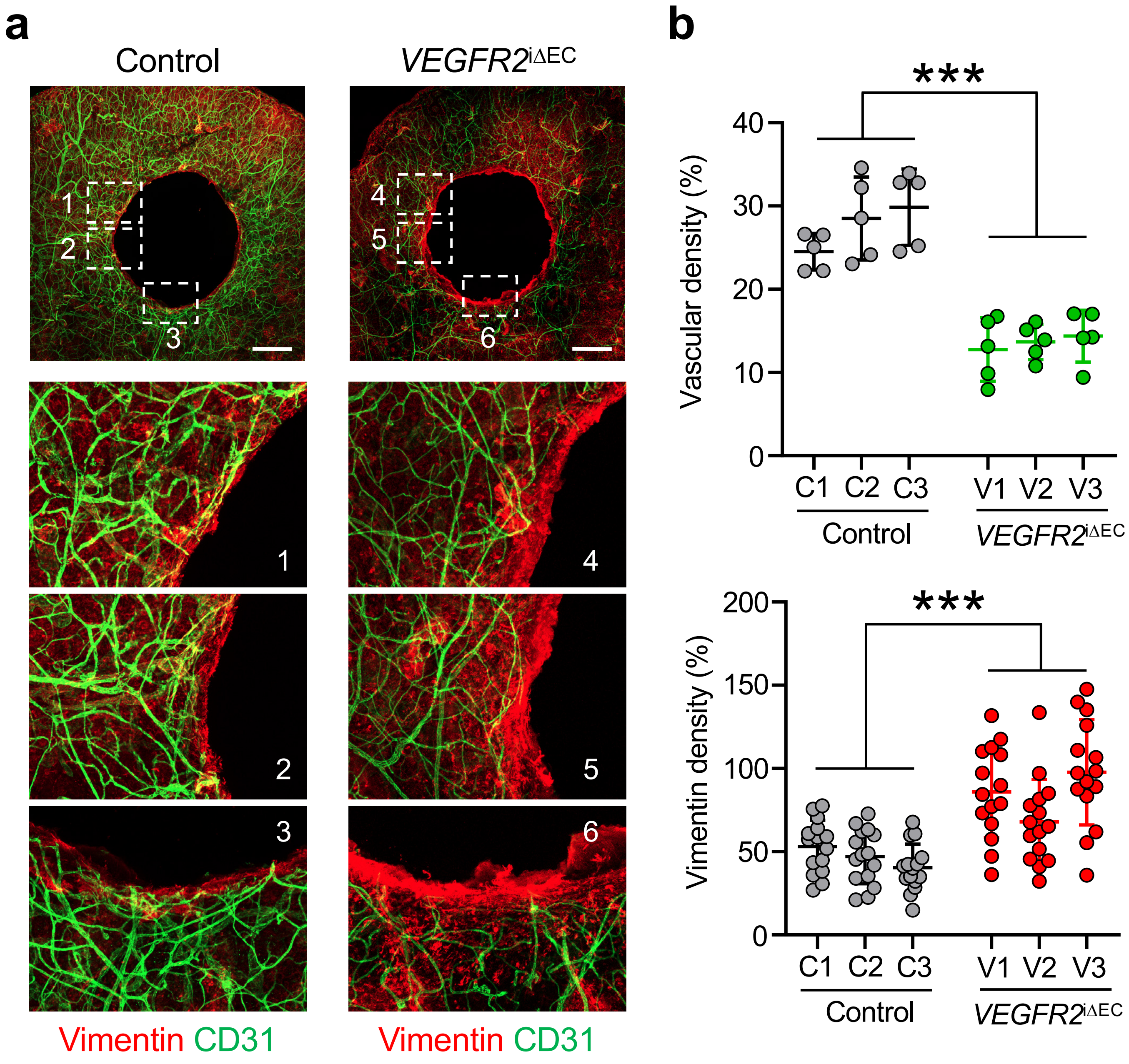

Supplementary Figure 14. Failure of vascular regeneration leads to accumulation of vimentin+ fibroblasts in the injury area of ear skin after punch-hole injury. a, **b**, Representative images and comparisons of the distribution of vimentin+ fibroblasts in the circumferential injury area of ear skin in Control and *VEGFR2*$^{i\Delta EC}$ mice at D14 after punch-hole injury. Each numbered box area is magnified in below 3 panels. Scale bars, 1.0 mm. 5 values were obtained from each mouse for vascular density, while 15 values were obtained from each mouse for vimentin density. *n* = 3 mice (C1, C2, C3; V1, V2, V3)/group from two independent experiments. Vertical bars indicate mean ± SD. ****P* <0.001 versus Control by two-tailed multiple group comparison t-test.

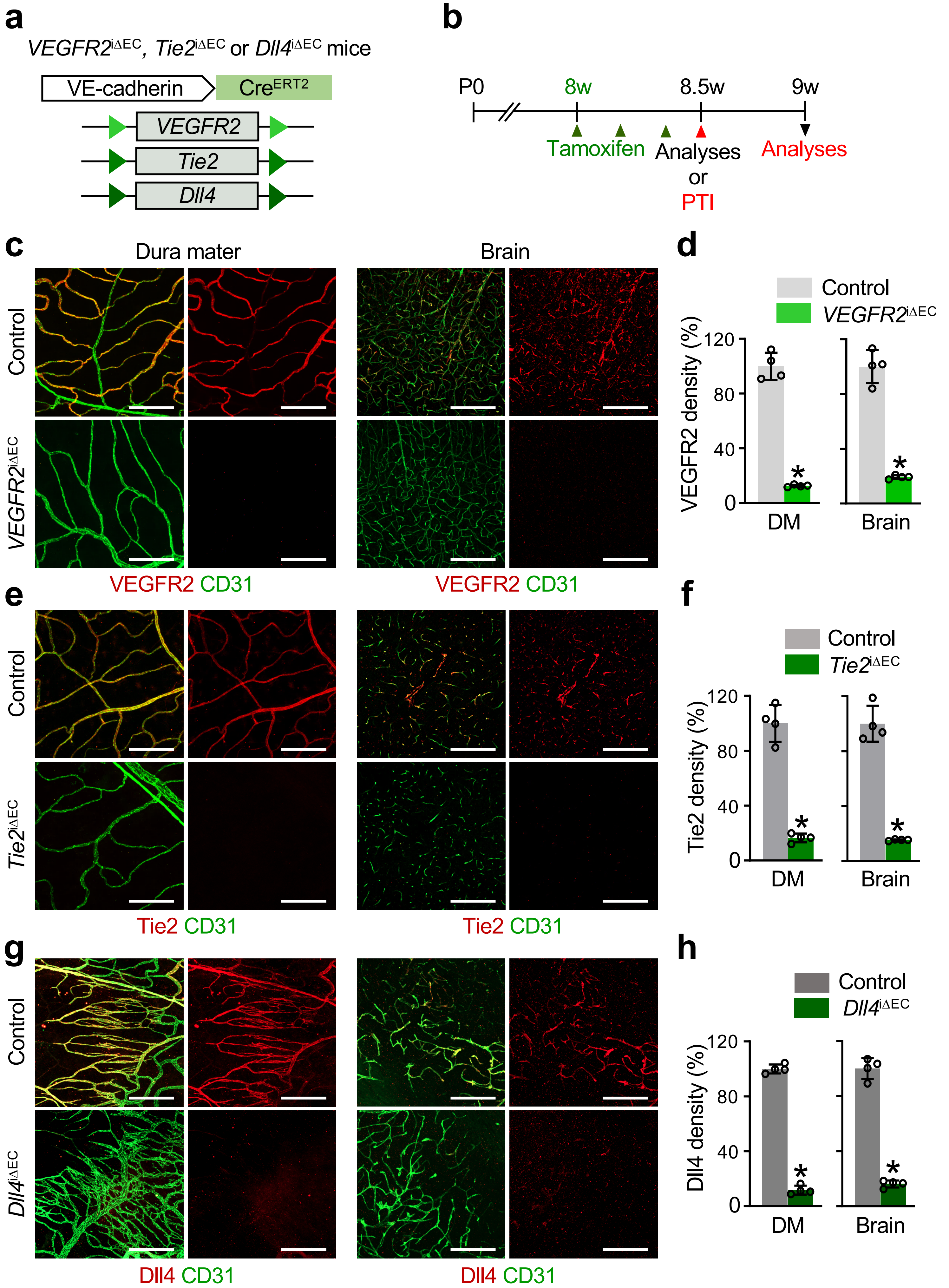
a
VEGFR2iΔEC, Tie2iΔEC or Dll4iΔEC mice
VE-cadherin
CreERT2
VEGFR2
Tie2
Dll4
b
P0
8w
8.5w
9w
Tamoxifen
Analyses
or
PTI
Analyses
c
Dura mater
Brain
Control
VEGFR2iΔEC
VEGFR2 CD31
VEGFR2 CD31
d
Control
VEGFR2iΔEC
VEGFR2 density (%)
120
80
40
0
DM
Brain
e
Control
Tie2iΔEC
Tie2 CD31
Tie2 CD31
f
Control
Tie2iΔEC
Tie2 density (%)
120
80
40
0
DM
Brain
g
Control
Dll4iΔEC
Dll4 CD31
Dll4 CD31
h
Control
Dll4iΔEC
Dll4 density (%)
120
80
40
0
DM
Brain

Supplementary Figure 15. Effective deletion of indicated receptors in blood vessels of both dura mater and brain using *VEGFR2*$^{i\Delta EC}$, *Tie2*$^{i\Delta EC}$ or *Dll4*$^{i\Delta EC}$ mice.
a,**b**, Diagram depicting generation of *VEGFR2*$^{i\Delta EC}$, *Tie2*$^{i\Delta EC}$ or *Dll4*$^{i\Delta EC}$ mice and EC-specific deletion of *VEGFR2*, *Tie2* or *Dll4* in 8-week-old mice by i.p. injections of tamoxifen, PTI, and their analyses at 3 days or 1 week after PTI. **c-h**, Representative images and comparisons of densities of VEGFR2, Tie2 and Dll4 between Control and *VEGFR2*$^{i\Delta EC}$ or *Tie2*$^{i\Delta EC}$ mice at 3 days after tamoxifen or *Dll4*$^{i\Delta EC}$ at 3 days after PTI. Scale bars, 200 μm. Each dot indicates a value from one mouse and n = 4 mice/group from two independent experiments. Vertical bars indicate mean ± SD. *P = 0.0145 versus Control by two-tailed Mann-Whitney U test. DM, dura mater.
