# Supplementary material for: VEGFR2 signaling drives meningeal vascular regeneration upon head injury
Source: Nat Commun. 2020 Jul 31;11:3866. doi: 10.1038/s41467-020-17545-2 (PMC7395111; doi:10.1038/s41467-020-17545-2)
Supplement: Supplementary file 2 — Reporting Summary [file 41467_2020_17545_MOESM2_ESM.pdf]

nature research

Corresponding author(s): Gou Young Koh and Injune Kim

Last updated by author(s): May 20, 2020

Reporting Summary

Nature Research wishes to improve the reproducibility of the work that we publish. This form provides structure for consistency and transparency in reporting. For further information on Nature Research policies, see Authors & Referees and the Editorial Policy Checklist.

Statistics

For all statistical analyses, confirm that the following items are present in the figure legend, table legend, main text, or Methods section.

n/a	Confirmed	
☐	☒	The exact sample size (*n*) for each experimental group/condition, given as a discrete number and unit of measurement
☐	☒	A statement on whether measurements were taken from distinct samples or whether the same sample was measured repeatedly
☐	☒	The statistical test(s) used AND whether they are one- or two-sided<br>*Only common tests should be described solely by name; describe more complex techniques in the Methods section.*
☐	☒	A description of all covariates tested
☐	☒	A description of any assumptions or corrections, such as tests of normality and adjustment for multiple comparisons
☐	☒	A full description of the statistical parameters including central tendency (e.g. means) or other basic estimates (e.g. regression coefficient) AND variation (e.g. standard deviation) or associated estimates of uncertainty (e.g. confidence intervals)
☐	☒	For null hypothesis testing, the test statistic (e.g. *F*, *t*, *r*) with confidence intervals, effect sizes, degrees of freedom and *P* value noted<br>*Give P values as exact values whenever suitable.*
☒	☐	For Bayesian analysis, information on the choice of priors and Markov chain Monte Carlo settings
☒	☐	For hierarchical and complex designs, identification of the appropriate level for tests and full reporting of outcomes
☒	☐	Estimates of effect sizes (e.g. Cohen's *d*, Pearson's *r*), indicating how they were calculated

Our web collection on statistics for biologists contains articles on many of the points above.

Software and code

Policy information about availability of computer code

Data collection	LSM image software (Carl Zeiss)<br>Zen 2.3 software (Carl Zeiss)<br>CFX Manager 3.0 software (Bio-Rad)<br>FACS DIVA v8.0.1 (BD)
Data analysis	Zen 2.3 software (Carl Zeiss)<br>ImageJ 1.8.0 software (NIH)<br>CFX Manager 3.0 software (Bio-Rad)<br>GraphPad Prism 8.0 (GraphPad Software)<br>TopHat 2.1.1 software (Johh Hopkins University Center for Computational Biology)<br>Ingenuity Pathway Analysis 2.3 software (Qiagen)<br>Cell Ranger 3.1.0 toolkit (10X Genomics)<br><br>For single cell data, we used publicly available R packages:<br>Seurat (version 3.1.0)

For manuscripts utilizing custom algorithms or software that are central to the research but not yet described in published literature, software must be made available to editors/reviewers. We strongly encourage code deposition in a community repository (e.g. GitHub). See the Nature Research guidelines for submitting code & software for further information.

Data

Policy information about availability of data

All manuscripts must include a data availability statement. This statement should provide the following information, where applicable:

- Accession codes, unique identifiers, or web links for publicly available datasets
- A list of figures that have associated raw data
- A description of any restrictions on data availability

RNA sequencing data are available in the National Center for Biotechnology Information's Gene Expression Omnibus under accession numbers GSE145973 (scRNAseq) and GSE138561 (bulk RNAseq). The source data underlying all Figs. and Supplementary Figs. are provided as a Source Data file. All other data that support the findings of this study are available from the corresponding author upon reasonable request.

Field-specific reporting

Please select the one below that is the best fit for your research. If you are not sure, read the appropriate sections before making your selection.

☒ Life sciences ☐ Behavioural & social sciences ☐ Ecological, evolutionary & environmental sciences

For a reference copy of the document with all sections, see nature.com/documents/nr-reporting-summary-flat.pdf

Life sciences study design

All studies must disclose on these points even when the disclosure is negative.

Sample size	Sample sizes were chosen on the basis of standard power calculations (with α=0.05 and power of 0.8) performed for similar experiments and statistical methods were not used to predetermine sample sizes.
Data exclusions	No samples were excluded from analysis.
Replication	Experiments were replicated at least once for all analyses and number of reproductions of each experimental finding is described in each figure legend. All attempts at experimental replication were successful.
Randomization	Animals from different cages, but within the same experimental group, were selected to assure randomization.<br>All human autopsy sample subjects, irrespective of gender and age, with visible injuries in both the dura mater and brain were analyzed and contralateral sides of these samples were used as controls.
Blinding	The investigators were blinded during the experiments and quantifications.

Reporting for specific materials, systems and methods

We require information from authors about some types of materials, experimental systems and methods used in many studies. Here, indicate whether each material, system or method listed is relevant to your study. If you are not sure if a list item applies to your research, read the appropriate section before selecting a response.

Materials & experimental systems

n/a	Involved in the study	
☐	☒	Antibodies
☐	☒	Eukaryotic cell lines
☒	☐	Palaeontology
☐	☒	Animals and other organisms
☐	☒	Human research participants
☒	☐	Clinical data

Methods

n/a	Involved in the study	
☒	☐	ChIP-seq
☒	☐	Flow cytometry
☒	☐	MRI-based neuroimaging

Antibodies

Antibodies used	The following primary antibodies were used in the immunostaining of mouse samples: anti-CD31 (hamster monoclonal, MAB1398Z, Merck), anti-pVEGFR2 (Tyr1175, rabbit monoclonal, 2478, Cell Signaling Technology), anti-pERK (Thr202/Tyr204, rabbit monoclonal, 4370, Cell Signaling Technology), anti-pAkt (Ser473, rabbit monoclonal, 4060, Cell Signaling Technology), anti-VE-PTP (rabbit polyclonal, generously provided by Dietmar Vestweber), anti-VEGFR2 (goat polyclonal, AF644, R&D), anti-VEGFR3 (goat polyclonal, AF743, R&D), anti-Tie2 (goat polyclonal antibody, AF762, R&D), anti-Dll4 (goat polyclonal, AF1389, R&D), anti-Ang246, anti-F4/80 (rat monoclonal, MCA497, Bio-Rad), anti-LYVE-1 (rabbit polyclonal, 11-034, Angiobio), anti-VEGF164 (goat polyclonal, AF-493-NA, R&D), anti-Osterix (rabbit polyclonal, ab22552, Abcam), anti-collagen type IV (rabbit polyclonal, ab6586, Abcam), anti-vimentin (chicken polyclonal, AB5733, Merck) and anti-neurofilament heavy polypeptide (rabbit polyclonal, ab8135, Abcam).

	The following primary antibodies were used in the immunostaining of human samples: anti-VEGFR2 (goat polyclonal, AF357, R&D) and anti-CD31 (rabbit polyclonal, ab28364, Abcam).<br><br>Alexa Fluor 488-conjugated goat anti-Armenian hamster (127-545-160), goat anti-rabbit (111-545-144), donkey anti-goat (705-545-147), goat anti-human (109-545-088), goat anti-rat (112-545-167); Alexa Fluor 594-conjugated goat anti-Armenian hamster (127-585-160), goat anti-rabbit (111-585-144), donkey anti-goat (705-585-147), goat anti-human (109-585-088), goat anti-rat (112-585-167), goat anti-chicken (103-585-155); Alexa Fluor 647-conjugated goat anti-hamster (127-605-160), goat anti-rabbit (111-605-144), goat anti-chicken (103-605-155) secondary antibodies were purchased from Jackson ImmunoResearch.<br><br>The following antibodies were used for MACS: biotin anti-mouse CD31 (rat-monoclonal, 130-119-562, Miltenyi-Biotec); anti-biotin microbeads (130-090-485, Miltenyi-Biotec).<br><br>The following antibodies were used for FACS: PE anti-mouse CD31 (rat monoclonal, 102508, Biolegend); APC anti-mouse CD45 (rat monoclonal, 103112, Biolegend); FITC anti-mouse TER-119 (rat monoclonal, 116206, Biolegend).
Validation	All the antibodies were validated for the species (mouse or human) and applications (immunohistochemistry) by the corresponding manufacturer, which is described in the manufacturer's website. Our usage is described in the Methods section of the manuscript as below:<br><br>After blocking with 5% goat or donkey serum (Jackson ImmunoResearch) in 0.3% Triton-X 100 in PBS (PBST) for 1 h, samples were incubated with the indicated primary antibodies diluted in the blocking solution at 4°C overnight. After several washes with PBST, the samples were incubated at 4°C overnight with the indicated fluorochrome-conjugated secondary antibodies diluted in the blocking buffer.

Eukaryotic cell lines

Policy information about cell lines

Cell line source(s)	DC101 hybridoma (ATCC HB-11534; ATCC; rat (B cell), mouse (myeloma); spleen)<br>APB5 hybridoma (Dr. Akiyoshi Uemura; Nagoya City University; rat (B cell), mouse (myeloma); spleen)
Authentication	Cells were authenticated based on morphology, growth conditions, and blocking antibodies purified from these cells lines were tested for specific binding/blocking efficiencies in both in vitro and in vivo settings.
Mycoplasma contamination	All cell lines tested negative for mycoplasma contamination.
Commonly misidentified lines<br>(See ICLAC register)	No commonly misidentified cell lines were used in this study.

Animals and other organisms

Policy information about studies involving animals; ARRIVE guidelines recommended for reporting animal research

Laboratory animals	Specific pathogen-free (SPF) C57BL/6J were purchased from DBL Co., Ltd. (Chung-cheong bukdo, Republic of Korea). VE-cadherin-CreERT2 (Okabe et al., Cell, 2014; provided by Dr. Yoshiaki Kubota, Keio University), VEGF-A+/LacZ (Miquerol et al., Dev Bio, 1999; provided by Dr. Hoon-Ki Sung, University of Toronto), Vegfr2-floxed (Sison et al., J Am Soc Nephrol., 2010; provided by Dr. Masanori Hirashima, Kobe University), Tie2-floxed (Savant et al., Cell Rep., 2015; provided by Dr. Hellmut Augustin, Heidelberg University), Dll4-floxed (Hozumi et al., J Exp Med., 2008; provided by Dr. David Shima, University College London), PDGFRb-Cre-ERT2 (Chen et al., Nat Commun., 2016; provided by Dr. Ralf Adams, Max Planck Institute, Muenster), Actin-GFP (Jackson Laboratory), Cx3cr1-DTR (Jackson Laboratory) and R26-tdTomato (Jackson Laboratory) mice were transferred, established, and bred in SPF animal facilities at KAIST. Cre-ERT2 negative but flox/flox positive mice among the littermates of these mice were defined as control (WT) mice. All of these mice were maintained in the C57BL/6 background. Adult male mice at 8~10 weeks of age were used for all experiments.<br>All mice were housed under 12 hr light/12 hr dark cycle, temperatures of 22±2°C with 50±10% humidity and fed with free access to a standard diet (PMI LabDiet) and water.
Wild animals	The study did not involve wild animals.
Field-collected samples	The study did not involve samples collected from the field.
Ethics oversight	Animal care and experimental procedures were performed under the approval from the Institutional Animal Care and Use Committee of KAIST (No. KA2018-42). All procedures and animal handlings were performed following the ethical guidelines for animal studies.

Note that full information on the approval of the study protocol must also be provided in the manuscript.

Human research participants

Policy information about studies involving human research participants

Population characteristics	2 males and 2 females, deceased at 30~50 years of age, with severe head injury from vertical falls. Past diagnoses and treatment are not applicable.

Recruitment	Recruitment protocol does not apply to this study, since autopsies were performed with court-issued warrants at the request of the public prosecutor of Chonnam Province, South Korea. All cases with severe head injury (visible injury to both dura mater and brain) were analyzed.
Ethics oversight	Institutional Review Board of Chonnam National University Medical School and Hospital (Gwangju, Republic of Korea) as research activity involving non-human participants (No. 2019-001)

Note that full information on the approval of the study protocol must also be provided in the manuscript.
